# Three Birds, One Stone: An Osteo‐Microenvironment Stage‐Regulative Scaffold for Bone Defect Repair through Modulating Early Osteo‐Immunomodulation, Middle Neovascularization, and Later Osteogenesis

**DOI:** 10.1002/advs.202306428

**Published:** 2023-12-07

**Authors:** Yuhao Yuan, Yan Xu, Yiyang Mao, Hongbin Liu, Minning Ou, Zhangyuan Lin, Ruibo Zhao, Haitao Long, Liang Cheng, Buhua Sun, Shushan Zhao, Ming Zeng, Bangbao Lu, Hongbin Lu, Yong Zhu, Can Chen

**Affiliations:** ^1^ Department of Orthopedics Xiangya Hospital Central South University Changsha Hunan 410008 China; ^2^ National Clinical Research Center for Geriatric Disorders Xiangya Hospital Central South University Changsha Hunan 410008 China; ^3^ Key Laboratory of Organ Injury Aging and Regenerative Medicine of Hunan Province Changsha Hunan 410008 China; ^4^ Department of Sports Medicine Xiangya Hospital Central South University Changsha Hunan 410008 China

**Keywords:** 3D‐printed scaffold, bone regeneration, decellularized bone matrix, magnesium hydroxide, osteo‐microenvironment

## Abstract

In order to repair critical‐sized bone defects, various polylactic acid‐glycolic acid (PLGA)‐based hybrid scaffolds are successfully developed as bone substitutes. However, the byproducts of these PLGA‐based scaffolds are known to acidify the implanted site, inducing tiresome acidic inflammation. Moreover, these degradation productions cannot offer an osteo‐friendly microenvironment at the implanted site, matching natural bone healing. Herein, inspired by bone microenvironment atlas of natural bone‐healing process, an osteo‐microenvironment stage‐regulative scaffold (P80/D10/M10) is fabricated by incorporating self‐developed decellularized bone matrix microparticles (DBM‐MPs) and multifunctional magnesium hydroxide nanoparticles (MH‐NPs) into PLGA with an optimized proportion using low‐temperature rapid prototyping (LT‐RP) 3D‐printing technology. The cell experiments show that this P80/D10/M10 exhibits excellent properties in mechanics, biocompatibility, and biodegradability, meanwhile superior stimulations in osteo‐immunomodulation, angiogenesis, and osteogenesis. Additionally, the animal experiments determined that this P80/D10/M10 can offer an osteo‐friendly microenvironment in a stage‐matched pattern for enhanced bone regeneration, namely, optimization of early inflammation, middle neovascularization, and later bone formation. Furthermore, transcriptomic analysis suggested that the in vivo performance of P80/D10/M10 on bone defect repair is mostly attributed to regulating artery development, bone development, and bone remodeling. Overall, this study reveals that the osteo‐microenvironment stage‐regulative scaffold provides a promising treatment for bone defect repair.

## Graphical abstract

Inspired by the bone microenvironment atlas of natural bone healing, an osteo‐microenvironment stage‐regulative scaffold is printed by incorporating self‐developed DBM‐MPs and multifunctional MH‐NPs into PLGA with an optimized proportion. This scaffold is suitable as a bone substitute for filling bone defects. More importantly, as degradation, it can sequentially modulate early inflammation, middle vascularization, and later osteogenesis, thus enhancing bone regeneration.

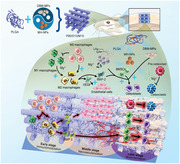

## 1 Introduction

Critical‐sized bone defects caused by trauma and other bone diseases are difficult to achieve functional restoration without surgical intervention.^[^ ^1^ ^]^ In clinics, autologous bone graft from a patient's ilium is regarded as the gold standard for bone defect treatment, but donor‐site morbidity, limited graft acquisition, and prolonged operation time impose constraints and make it unsuitable in many cases.^[^ ^2^ ^]^ To overcome these limitations, various 3D‐printed bone scaffolds have been developed as bone substitutes using 3D‐printing technology, which acts as a temporary matrix for bone formation.^[^ ^3^ ^]^ However, the design of these 3D‐printed bone scaffolds has mainly focused on printability, mechanical strength, degradation rate, as well as osteogenic and/or angiogenic activities, but few considered their degradation productions‐induced local microenvironment on bone formation.^[^ ^1^, ^3^, ^4^ ^]^ Thus, next 3D‐printed bone scaffolds should be fabricated with optimal biomaterials, thus not only functioning as a bone substitute but also continuously building an osteo‐friendly microenvironment for bone defect repair via degradation products.

Metals, bioceramics, and synthetic polymers have been used to produce 3D‐printed bone scaffolds.^[^ ^1^, ^3^, ^5^ ^]^ However, most metals are undegradable, bioceramics are very brittle, and synthetic polymers show poor osteoinductive properties, more and more researchers favor blending metals and/or bioceramics into synthetic polymers to address their limitations. Polylactic acid‐glycolic acid (PLGA), as a biodegradable polymer, has been widely used in 3D‐printed bone scaffolds, due to its favorable biocompatibility and adjustable biodegradation.^[^ ^5^, ^6^ ^]^ Nevertheless, inherent shortcomings remain associated with PLGA including low osteoinductivity, limited mechanics, and acidic degradations‐induced inflammatory response.^[^ ^5^, ^6^, ^7^ ^]^ Currently, hydroxyapatite (HA) or tricalcium phosphate (TCP) has been successfully integrated into PLGA to improve its osteoinductive and mechanical properties.^[^ ^5^, ^8^ ^]^ However, the HA or the TCP was just an inorganic component in natural bone, without mimicking the original components and ultrastructure of natural bone extracellular matrix (ECM). Thus, finding a biomimetic material capable of replacing the HA or the TCP is needed and meaningful.

The decellularization technique has generated great interest in tissue engineering since it can fabricate a biomimetic material with low immunogenicity, high biocompatibility, good biodegradability, and excellent bioactivity.^[^ ^9^ ^]^ Nowadays, decellularized bone matrix (DBM), showing high similarities to natural bones in the mechanics, components, and ultrastructure, has been gradually used in bone tissue engineering.^[^ ^10^ ^]^ DBM was composed of HA mineralized collagen, glycoproteins, proteoglycans, cytokines, and growth factors, thus suitable for stem cell attachment and capable of inducing interacted stem cells differentiating into osteogenic lineage.^[^ ^10^ ^]^ Theoretically, it may be a biomimetic material for replacing the HA or the TCP in bone tissue engineering. Regrettably, DBMs exhibit a blocky appearance, which makes it impossible for blending within PLGA to print a 3D porous scaffold. To overcome this shortage, in this study, bulk DBM was novelty processed into the shape of microparticles. These microparticles of DBM (DBM‐MPs) preserved the biomimetic and osteogenic properties of blocky DBM.^[^ ^11^ ^]^ More importantly, DBM‐MPs exposed more collagen to these anchored cells than blocky DBM, more convenient for their biomineralization within and around collagen fibrils.^[^ ^11^ ^]^ Based on these advantages, the DBM‐MPs were selected as a replacement material for the HA or the TCP and blended into PLGA to improve its osteoinductive and mechanical properties.

Apart from the osteoinductive and mechanical properties, an ideal PLGA hybrid bone scaffold should have the potential for continuously building an osteo‐friendly microenvironment for bone defect repair via degradation products. Generally, bone repair sequentially undergoes early inflammation, middle fibrovascular emergence, late bone formation and remodeling, which are respectively associated with a set of finely coordinated local microenvironment changes,^[^ ^12^ ^]^ namely, macrophage polarization to pro‐healing M2 phenotypes in early inflammatory stage;^[^ ^13^ ^]^ neovascularization to supply oxygen and nutrients in middle fibrovascular stage^[^ ^14^ ^]^; controlling cellular functions of bone marrow mesenchymal stem cells (BMSCs), osteoblasts, and osteoclasts in late bone formation and remodeling phase.^[^ ^1^, ^15^ ^]^ Therefore, novel PLGA hybrid bone scaffolds should contain some multifunctional materials, that would sequentially modulate macrophage polarization, neovascularization, or osteogenic differentiation of BMSCs in a stage‐matched pattern as scaffold degradation.

In recent years, metallic magnesium (Mg) and magnesium ceramics have gained tremendous attention, and have been extensively investigated as multifunctional biomaterials in bone tissue engineering.^[^ ^16^ ^]^ Previous studies revealed that the mechanisms of metallic magnesium and magnesium ceramics on promoting bone regeneration include generating a pro‐osteogenic immune microenvironment by modulating macrophage polarization toward M2 phenotype;^[^ ^17^ ^]^ improving revascularization that transfers oxygen and nutrients for bone formation;^[^ ^14^, ^18^ ^]^ directly stimulating osteogenic differentiation of BMSCs and inhibiting osteoclast activity.^[^ ^16^, ^19^ ^]^ These revealed mechanisms indicate that metallic magnesium and magnesium ceramics may be the required multifunctional biomaterial. Nevertheless, inherent deficiencies remain associated with metallic magnesium, including too fast degradation rate, production of hydrogen gas leading to tissue necrosis, and difficulty in manufacturing a porous or shape‐matching scaffold.^[^ ^16a,d^ ^]^ Therefore, magnesium ceramics have become the additive of choice in biomaterials over the past decade. Magnesium hydroxide nanoparticles (MH‐NPs), as a commonly used magnesium ceramic in bone tissue engineering, proved to have multi‐modulation on macrophage polarization, revascularization, and osteogenesis, meanwhile have been utilized to offset the degraded acidic byproducts from PLGA and improve the mechanical strength of PLGA.^[^ ^7^, ^16^, ^20^ ^]^ Hence, MH‐NPs were added to the 3D‐printed PLGA hybrid bone scaffold to function as a stage‐matched regulator of the bone formation environment and a neutralizer of acidic inflammation along with scaffold degradation.

**Figure 1:**
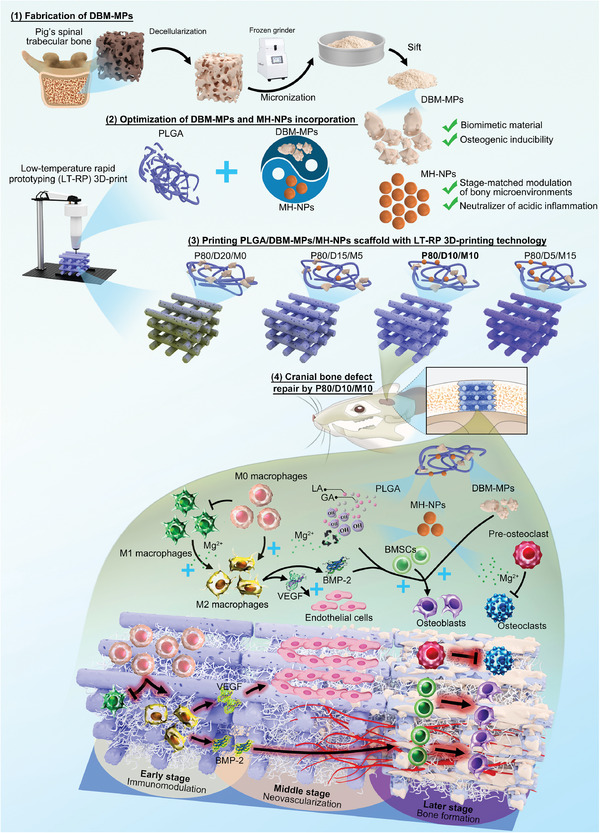
Schematic illustration of a novel 3D‐printed PLGA/DBM‐MPs/MH‐NPs scaffold (P80/D10/M10) for the enhancement of endogenous bone regeneration. Firstly, DBM‐MPs were novelty developed; Secondly, using an optimized proportion, a 3D‐printing ink was prepared by incorporating 10% DBM‐MPs and 10% MH‐NPs into PLGA; Thirdly, LT‐RP 3D‐printing technology was used to print PLGA/DBM‐MPs/MH‐NPs scaffold; Lastly, the P80/D10/M10 offer an osteo‐friendly microenvironment in a stage‐matched pattern for enhanced bone regeneration, namely, optimization of early inflammation, middle neovascularization, and later bone formation.

As shown in **Figure** **1**, in order to provide a stage‐matched osteo‐friendly microenvironment for bone defect repair, two biocompatible, biodegradable, and multifunctional materials (DBM‐MPs and MH‐NPs) were integrated into PLGA with an optimized proportion as a novel 3D‐printing ink, to avoid the inactivation of inherent proteins in DBM‐MPs, low‐temperature rapid prototyping (LT‐RP) 3D‐printing technology was applied for printing the PLGA/DBM‐MPs/MH‐NPs scaffold, thus ensuring its superior mechanical, osteoinductive, osteoconductive properties. The gradually exposed DBM‐MPs as scaffold degradation could provide space for stem cell anchorage while efficiently guiding them to differentiate into osteogenic linage. In addition, MH‐NPs could neutralize the acidic products of PLGA degradation, meanwhile continuously releasing Mg ions to modulate early macrophage polarization, middle neovascularization, and later osteogenesis. This study may design an advanced PLGA hybrid bone scaffold to overcome the disadvantages of conventional PLGA hybrid bone scaffolds and effectively induce bone defect repair by stage‐matched orchestrating osteo‐microenvironment.

## 2 Results

### 2.1 Fabrication of PLGA/DBM‐MPs/MH‐NPs Scaffolds

**Figure 2:**
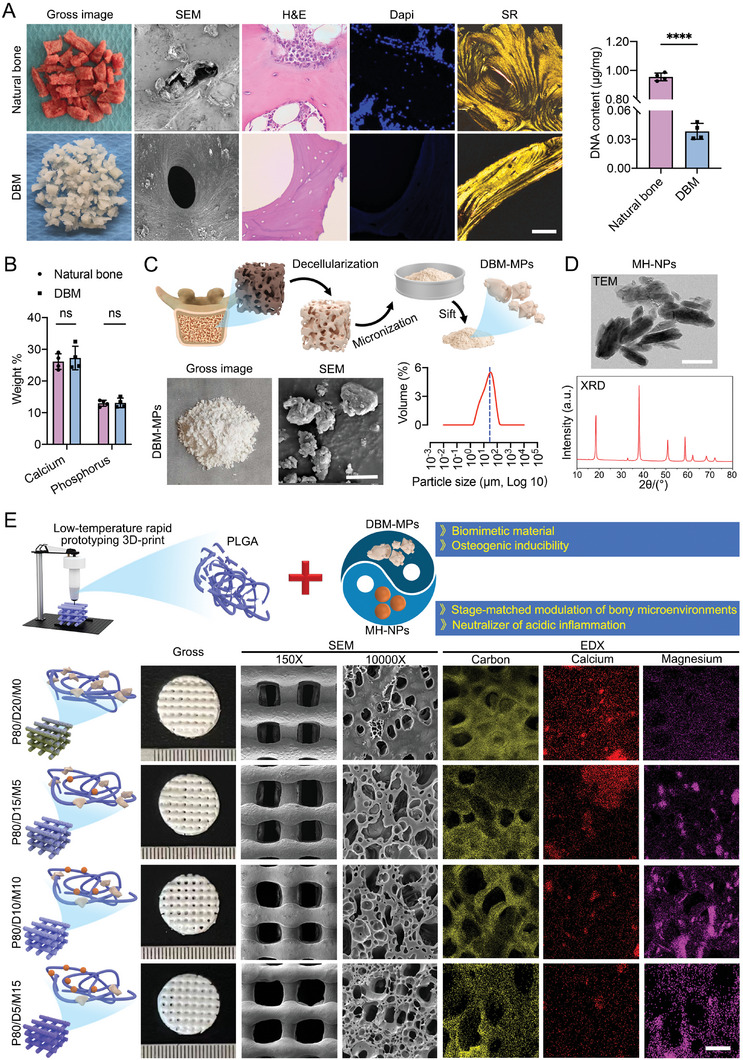
Fabrication of 3D‐printed PLGA/DBM‐MPs/MH‐NPs scaffolds. A) SEM, H&E, DAPI, and SR (Sirius red) staining were used to evaluate the morphological changes of the bone matrix before and after decellularization, and DNA residues were detected. Scale bar = 50 µm. B) Content of calcium (Ca) and phosphorus (P) in DBM‐MPs and normal bone evaluated by EDS analysis. C) Gross image, SEM images, and particle size distribution of DBM‐MPs. Scale bar = 25 µm. D) TEM image and XRD pattern of MH‐NPs. Scale bar = 50 nm. E) Four kinds of scaffolds with different proportions of PLGA, DBM‐MPs, and MH‐NPs were fabricated by LT‐RP 3D‐printing technology. The microstructure of each scaffold was observed by SEM, and the distribution of carbon, calcium, and magnesium was evaluated by EDS analysis. Scale bar = 10 µm. Data are presented as mean ± SD (*n* = 4). *p*‐values are calculated using the unpaired *t*‐test. ^*^ *p* < 0.0332, ^**^ *p* < 0.0021, ^***^ *p* < 0.0002, ^****^ *p* < 0.0001, and “ns” indicates no significance.

After the trabecular bone blocks were dissected from the pig's spinal vertebrae at a local slaughterhouse, they were decellularized with our previous protocol.^[^ ^11^ ^]^ Morphologically, these DBM blocks showed a white surface in color (**Figure** **2**A); SEM images revealed that the DBM blocks presented similar ultrastructure with normal bone; In the hematoxylin and eosin (H&E) and DAPI‐stained images, DBM blocks contained no cellular substances, while SR‐stained images captured under polarized light showed that the collagen distribution and content in the DBM were similar to normal bone. Quantitatively, the DNA content in the DBM was lower than the level of inducing immune rejection (Figure 2A). EDS analysis determined that the content of calcium (Ca) and phosphorus (P) in the DBM blocks was similar to normal bone (Figure 2B; Figure S1, Supporting Information).

After that, the DBM blocks were ground into powder under low temperatures, these powders were sifted through steel wire sieves with an aperture of 74 µm to get DBM‐MPs (Figure 2C). As shown in Figure 2C, these DBM‐MPs showed an irregular shape with a bumpy surface under the observation of SEM. After analyzing the particle size, the mean volume of DBM‐MPs was 27.606 µm^3^, and the particle size uniformity of these DBM‐MPs was 4.587 µm at 10%, 20.485 µm at 50%, and 61.247 µm at 90%, indicating that that the sizes of DBM‐MPs prepared in our study were relatively uniform. In addition, live/dead stain and CCK‐8 assay showed that DBM‐MPs also have great biocompatibility (Figure S2, Supporting Information). The needle‐like morphology of MH‐NPs was observed, with a size of 10–20 nm in the short axis and 50–100 nm in the long axis. The XRD pattern shows a typical diffraction peak of MH‐NPs crystal (Figure 2D).

As shown in Figure 2E, using the LT‐RP 3D‐printing technology, four kinds of PLGA hybrid scaffolds were fabricated by incorporating different weight of DBM‐MPs and/or MH‐NPs (P80/D20/M0: 80% PLGA incorporated 20% DBM‐MPs; P80/D15/M5: 80% PLGA incorporated 15% DBM‐MPs and 5% MH‐NPs; P80/D10/M10: 80% PLGA incorporated 10% DBM‐MPs and 10% MH‐NPs; P80/D5/M15: 80% PLGA incorporated 5% DBM‐MPs and 15% MH‐NPs). The four different ratios of DBM‐MPs and/or MH‐NPs in PLGA were based on several similar studies.^[^ ^16^, ^20^ ^]^ The four kinds of PLGA hybrid scaffolds showed a homogeneous porous structure and were 3D‐printed with a pore diameter of 450–500 µm and a filament diameter of 300–350 µm. SEM images showed that the pores of these PLGA hybrid scaffolds showed an interconnected structure both horizontally and vertically. On the SEM images with high magnification, DBM‐MPs and MH‐NPs were covered by PLGA; Meanwhile, along with the increasing of MH‐NPs incorporation, the air bubbles inside the PLGA hybrid scaffold presented an increasing tendency, which is more convenient for cell adhesion and migration as well as oxygen and nutrients exchange; In addition, SEM‐EDS analysis determined that the P80/D20/M0 showed the highest calcium content, while the P80/D5/M15 had the highest magnesium content.

### 2.2 Physical Characterization of PLGA/DBM‐MPs/MH‐NPs Scaffolds

**Figure 3:**
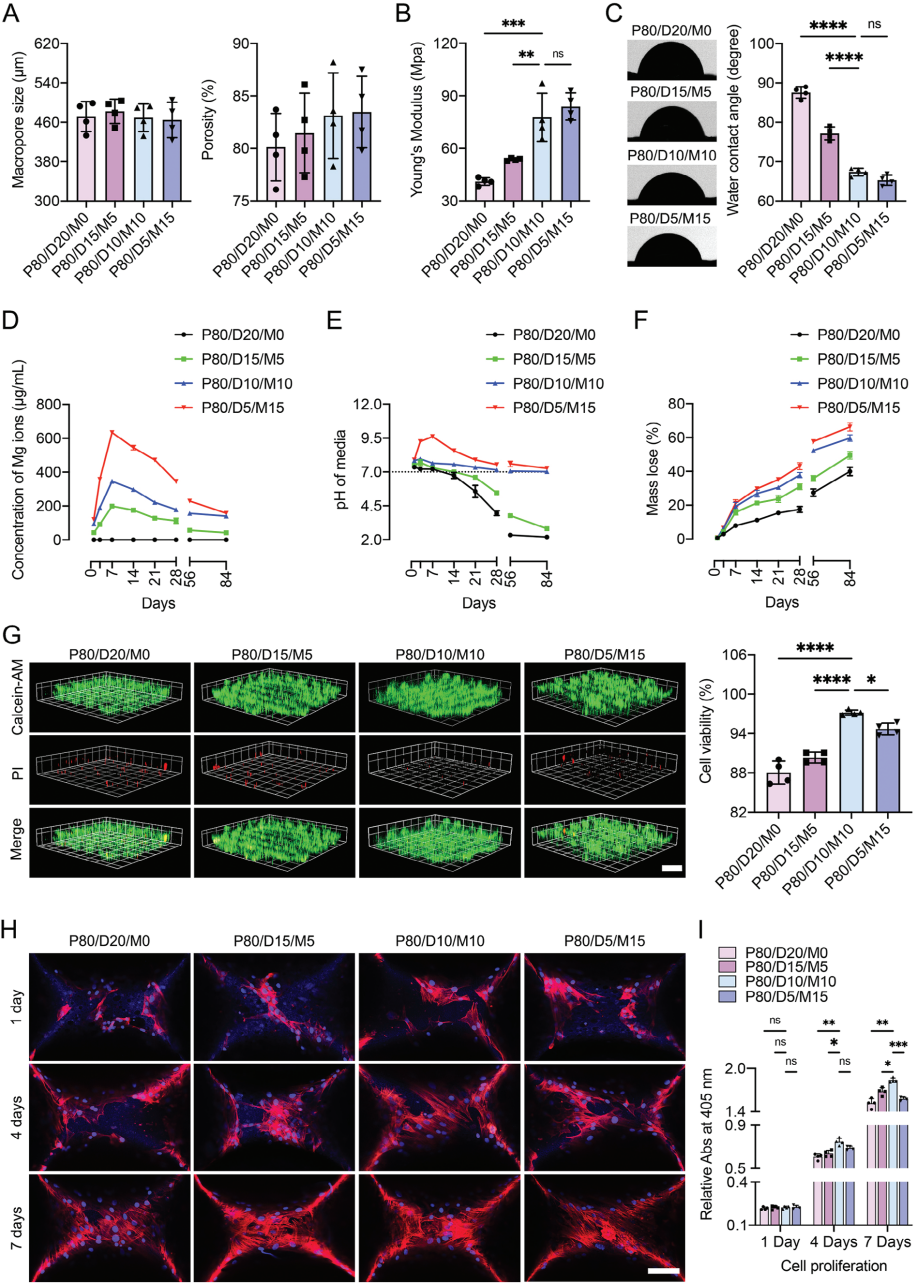
Physical characterization and cytocompatibility of 3D‐printed PLGA/DBM‐MPs/MH‐NPs scaffolds. A) Macropore size and porosity of scaffolds. B) Young's Modulus of scaffolds. C) Water contact angle measurement. D) In vitro release curve of Mg ions from PLGA hybrid scaffolds. E) pH changes of degradation medium and F) degradation curves for the four PLGA hybrid scaffolds. G) Cell viability of the four PLGA hybrid scaffolds using live/dead staining, 3D images show live (green) and dead (red) BMSCs in the scaffolds, and the rate of live cells to total cells was calculated as cell viability. Scale bar = 1 mm. H) Confocal laser microscopy images of the phalloidin/DAPI staining of BMSCs cultured on the scaffold at 1, 4, and 7 days. Scale bar = 200 µm. I) On days 1, 4, and 7, the CCK‐8 assay was used to assess the BMSCs proliferation on the four scaffolds. Data are presented as mean ± SD (*n* = 4). *p*‐values are calculated using one‐way ANOVA with Tukey's post hoc test. ^*^ *p* < 0.0332, ^**^ *p* < 0.0021, ^***^ *p* < 0.0002, ^****^ *p* < 0.0001, and “ns” indicates no significance.

Under SEM images, the macropore size of the four PLGA hybrid scaffolds was 471.6 ± 30.4 µm (P80/D20/M0), 482.2 ± 24.6 µm (P80/D15/M5), 469.4 ± 28.1 µm (P80/D10/M10), and 464.8 ± 35.9 µm (P80/D5/M15), respectively (**Figure** **3**A). The porosity of the four PLGA hybrid scaffolds was determined by the ethanol immersion method referenced in our previous protocol.^[^ ^21^ ^]^ The porosity of four PLGA hybrid scaffolds were 80.1 ± 3.2% (P80/D20/M0), 81.5 ± 3.8% (P80/D15/M5), 83.1 ± 4.1% (P80/D10/M10), and 83.5 ± 3.4% (P80/D5/M15), respectively (Figure 3A). There was no significant difference in the macropore size and the porosity among the four PLGA hybrid scaffolds.

With the increase of incorporated MH‐NPs, the PLGA hybrid scaffold showed a gradual improvement in mechanical strength. The Young's modulus of P80/D5/M15 and P80/D10/M10 were 83.9 ± 7.8 and 77.7 ± 13.7 MPa, which were higher than that of P80/D20/M0 (41.1 ± 2.2 MPa) and P80/D15/M5 (53.7 ± 0.8 MPa) (Figure 3B).

The surface wettability of the four kinds of PLGA hybrid scaffolds was evaluated to confirm the improved hydrophilicity. The water contact angle of those PLGA hybrid scaffolds decreased from 87.5° to 65.3° by incorporating DBM‐MPs and MH‐NPs (Figure 3C). The hydrophilic property of the scaffold directly determines the cell affinity and the exchange of culture media in cell‐related applications for tissue engineering.^[^ ^22^ ^]^

During degradation in vitro, the four kinds of PLGA hybrid scaffolds did not degrade completely at the end of 12 weeks (84 days). Meanwhile, we also analyzed the level of Ca ions and Mg ions in the degradation medium by inductively coupled plasma‐optical emission spectrometer (ICP‐OES) for 12 weeks. The results showed that the Ca ions detected in the degradation medium presented a very low level, indicating that the DBM‐MPs kept a steady state and did not release Ca ions rapidly (Figure S3, Supporting Information). The Mg ion releasing from P80/D15/M5, P80/D10/M10, P80/D5/M15 were various to different MH‐NPs portions. As shown in Figure 3D, the more MH‐NPs were incorporated into the PLGA hybrid scaffold, the faster Mg release from the scaffolds. Mg in the P80/D15/M5, P80/D10/M10, and P80/D5/M15 released rapidly in the early 7 days and then slowed down the release amount from day 7 to 28, finally reached a sustained rate from day 28 to 84.

According to published literature,^[^ ^23^ ^]^ MH‐NPs change into an unstable state in Phosphate buffered saline (PBS) solution and or body fluid and gradually release OH^−^ ions together with Mg ions. These released OH^−^ ions would function as a neutralizer for the acidic byproduct of PLGA and an enhancer for the degradation of PLGA hybrid scaffold. Thus, our in vitro experiment selected the PBS solution at 37 °C and 100 rpm wave as an imitation of in vivo body environment to evaluate the neutralizing effect of MH‐NPs and DBM‐MPs incorporated in PLGA scaffolds. As shown in Figure 3E, the pH of the media changed abruptly for the first 7 days, and the pH changes at the end of the 12 weeks ranged from 2.12 without MH‐NPs to 7.01 with neutralization of MH‐NPs. In the P80/D20/M0 group, the decrease in pH of the medium started on day 7, and it reached ≈2.20 at day 84. While, in the P80/D15/M5 group, a decrease in pH value was delayed up to day 14. On the other hand, for the P80/D5/M15 group, the pH level initially increased to up to 9.61 for the first 7 days and kept at above 7 during the rest period. As for the P80/D10/M10 group, the pH change behavior was relatively flat, which was attributed to the effect of dual neutralization in DBM‐MPs and MH‐NPs. The pH level of medium in the P80/D10/M10 group increased to 7.96 for the first 3 days and then stayed above 7 during the rest period. Thus, the gently neutralized scaffold may provide a desirable environment for cellular attachment and activities.

The degradation rate is another critical property of PLGA hybrid scaffolds, which directly influences the replacement of newly‐formed bone. Herein, the degradation rate of the four PLGA hybrid scaffolds was evaluated by assessing their weight in PBS solution at 37 °C at 100 rpm. As shown in Figure 3F, weight loss of the scaffolds was more significant in MH‐NPs‐incorporated groups than in the other groups, and the DBM‐MPs in the scaffolds were exposed more obviously (Figure S4, Supporting Information). Furthermore, the P80/D10/M10 group or P80/D5/M15 group demonstrated ≈59.68% or 66.23% weight loss at day 84, while the P80/D20/M0 group or P80/D15/M5 group showed 39.95% or 49.47% weight loss. These results imply that P80/D10/M10 or P80/D5/M15 have better potential regarding biodegradable materials than the P80/D10/M10 or the P80/D5/M15.

### 2.3 Cytocompatibility of PLGA/DBM‐MPs/MH‐NPs Scaffolds

The cytocompatibility of the P80/D20/M0, P80/D15/M5, P80/D10/M10, or P80/D5/M15 were examined with BMSCs viability, adhesion, and proliferation on their surface. As shown in Figure 3G, the Live/Dead assay showed that most of the BMSCs cultured with the P80/D20/M0, P80/D15/M5, P80/D10/M10, or P80/D5/M15 were stained by fluorescent green (living cells), with few red (dead cells). Quantitatively, cell viability of P80/D10/M10 and P80/D5/M15 were 97.17% and 94.69%, which were significantly larger than that of P80/D20/M0 (88.05%) and P80/D15/M5 (90.33%), implying that the P80/D10/M10 or the P80/D5/M15 are a favorable support for good cell viability.

In order to investigate the morphology of BMSCs adhering to the P80/D20/M0, P80/D15/M5, P80/D10/M10, or P80/D5/M15, BMSCs cultured on the scaffolds for 1, 4, and 7 days were stained with DAPI and phalloidin. As shown in Figure 3H, more BMSCs spread and grew along the surface of P80/D10/M10 showing healthy striation of cytoskeleton when compared with the P80/D20/M0, P80/D15/M5, or P80/D5/M15.

Additionally, the degree of BMSCs proliferation on the four scaffolds was assessed at 1, 4, and 7 days using CCK‐8 assay. As shown in Figure 3I, the number of BMSCs increased with the culture period, and improvement of BMSCs proliferation was also more noticeable in the P80/D10/M10 group than the P80/D20/M0, P80/D15/M5, and P80/D5/M15 groups; moreover, level of BMSCs proliferation was always highest on the P80/D10/M10 group. The hydrophilic surface properties and relatively neutral microenvironment due to MH‐NPs and DBM‐MPs incorporation might improve initial cell adhesion and growth on the PLGA hybrid scaffold.^[^ ^24^ ^]^ Moreover, the Mg ions released from the scaffold also played a beneficial role in regulating cell metabolism, then enhancing cell proliferation.^[^ ^25^ ^]^ These results indicated that the P80/D10/M10 is desirable for cell adhesion and proliferation.

### 2.4 In Vitro Optimization of Immune Inflammatory Response

Being an excellent bone substitute for bone defect repair, it should have low immunogenicity, and even could directly optimize the local immune environment at the early stage of bone defect repair, thus creating an accelerating effect for the following repair process. Macrophages play a critical role in regulating the local immune environment, and their higher plasticity makes them ideal candidates for developing immunomodulatory scaffolds.^[^ ^26^ ^]^ Thus, an ideal 3D‐printed PLGA/DBM‐MPs/MH‐NPs scaffold should have the ability to induce macrophages to polarize toward the M2 phenotype rather than the M1 phenotype, thus creating a favorable osteo‐immunomodulatory microenvironment by the secretion of inflammatory, angiogenic and osteogenic cytokines.^[^ ^12^, ^27^ ^]^

In our study, monocytes/macrophages (RAW 264.7 cells) were cultured with the four kinds of PLGA hybrid scaffolds, cell culture plate (CCP), or 1 µg mL^−1^ lipopolysaccharide (LPS). The RAW 264.7 cells cultured with CCP were set as negative control, while incubated with LPS to induce inflammatory conditions as positive control. In order to evaluate the modulative property of our PLGA hybrid scaffold on macrophage polarization, the expression of M1 markers (INOS and CD86) and M2 markers (CD163 and CD206) in the RAW 264.7 cells were evaluated. Moreover, the supernatants of RAW 264.7 cells were collected for detecting the pro‐inflammatory cytokines (TNF‐α, IL‐6, and IL‐1β) and anti‐inflammatory cytokine (IL‐10 and IL‐4) to evaluate the immunomodulatory activity of our PLGA hybrid scaffolds. In addition, BMP‐2 and VEGF‐α in the supernatants, two cytokines favorable for vascularization and osteogenesis, were also evaluated.

**Figure 4:**
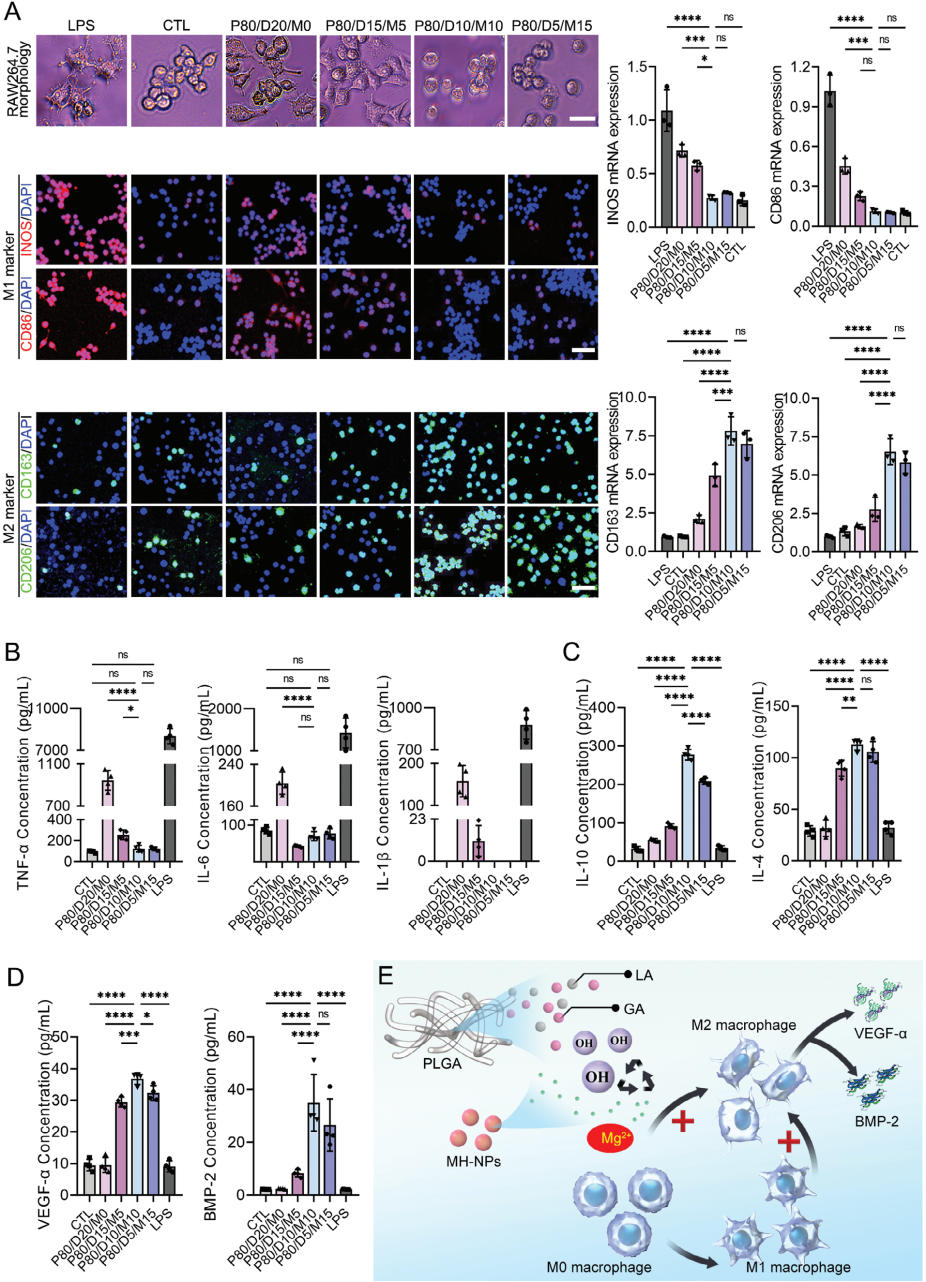
In vitro evaluation of immune inflammation of the four PLGA hybrid scaffolds. A) Microscope images of RAW 264.7 cells, expression levels of M1 (INOS and CD86) and M2 (CD163 and CD206) phenotypic markers in RAW264.7 cells under the stimulation of different PLGA hybrid scaffolds. Scale bar = 25 or 50 µm. B,C) The contents of pro‐inflammatory cytokines (TNF‐α, IL‐6, and IL‐1β) and anti‐inflammatory cytokines (IL‐10 and IL‐4) in the supernatants of RAW 264.7 cells under the stimulation of different PLGA hybrid scaffolds. D) The contents of VEGF‐α and BMP‐2 secreted by RAW 264.7 cells under scaffold stimulation. E) Schematic illustration showing the mechanisms of counteract acidic byproducts and immunomodulation by the incorporated MH‐NPs of PLGA hybrid scaffold. Data are presented as mean ± SD (*n* = 4). *p*‐values are calculated using one‐way ANOVA with Tukey's post hoc test. ^*^ *p* < 0.0332, ^**^ *p* < 0.0021, ^***^ *p* < 0.0002, ^****^ *p* < 0.0001, and “ns” indicates no significance.

As shown in **Figure** **4**A, most of RAW 264.7 markedly showed cell tentacles under the stimulation of LPS, lots of RAW 264.7 still exhibit cell tentacles cultured with the P80/D20/M0 and the P80/D15/M5, while no obvious cell tentacles were presented in the RAW 264.7 cultured with the CTL, P80/D10/M10 or P80/D5/M15. With the increase of MH‐NPs incorporation into the PLGA hybrid scaffold, the expression level of M1 markers (INOS and CD86) in the RAW 264.7 cells was gradually suppressed, meanwhile, the RAW 264.7 cells in the CTL, P80/D10/M10, and P80/D5/M15 groups showed significantly lower expression of INOS and CD86 than the P80/D20/M0, P80/D15/M5 and LPS groups, and no significant differences were found among the CTL, P80/D10/M10 and P80/D5/M15 groups. For the expression of M2 markers (CD163 and CD206) in the RAW 264.7 cells, the P80/D10/M10 group showed the highest expression level among the six groups.

As shown in Figure 4B, the pro‐inflammatory cytokines (TNF‐α and IL‐6) secreted by RAW 264.7 in the CTL, P80/D10/M10, or P80/D5/M15 group were similar without significant differences, but significantly lower than that of LPS, P80/D20/M0, or P80/D15/M5 group. Especially, IL‐1β secreted by RAW 264.7 in the CCP, P80/D10/M10, or P80/D5/M15 group were undetectable. As for the anti‐inflammatory cytokines (IL‐10 and IL‐4), the supernatants from the P80/D10/M10 group contain significantly higher levels of IL‐10 and IL‐4 than the other group (Figure 4C). In addition, the RAW 264.7 cells under the stimulation of P80/D10/M10 secret more VEGF‐α and BMP‐2 into cell supernatants in comparison with the RAW 264.7 cells cultured with the other scaffold (Figure 4D).

Those results indicated that, compared with the other PLGA hybrid scaffolds, the macrophages under the stimulation of P80/D10/M10 tend to polarize into the M2 phenotype rather than the M1 phenotype, and secret more anti‐inflammatory, angiogenic, and osteogenic cytokines (Figure 4E).

### 2.5 In Vitro Enhancement of Angiogenesis

Following the early inflammatory stage, the blood vessels will gradually invade the scaffold to eliminate metabolic waste and transmit oxygen and nutrients, which are important for the later osteogenesis process. Thus, an implant with good angiogenesis is a critical property in the middle stage of bone defect repair. In our study, human umbilical vein endothelial cells (HUVECs) were used to explore the angiogenic capacity of the four kinds of PLGA hybrid scaffolds using proliferation, migration, and tube formation experiments.

As shown in Figure S5 (Supporting Information), the P80/D10/M10 group shows the best HUVEC proliferation on day 1 after seeding due to the excellent cytocompatibility. After culturing for 5 days, the HUVECs proliferation ranking of different samples is P80/D20/M0 < CTL < P80/D15/M5 < P80/D5/M15 < P80/D10/M10. This result indicated that multifunctional Mg ions‐controlled release from the P80/D10/M10 scaffold has greatly facilitated the proliferation of HUVECs, which is attributed to the synergistic influence of the neutral environment and the stimulation of Mg ions.

**Figure 5:**
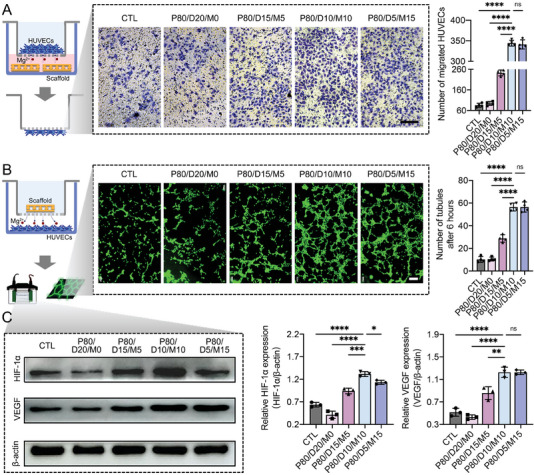
In vitro angiogenic capacity of the four PLGA hybrid scaffolds. A) Using Transwell assay to evaluate the HUVECs migration under the influence of different PLGA hybrid scaffolds. Scale bar = 200 µm. B) The effect of different PLGA hybrid scaffolds on tube formation of HUVECs at 6 h. Scale bar = 150 µm. C) Representative western‐blot images and semi‐quantitative data showing the expression of HIF1‐α and VEGF in HUVECs co‐cultured with the four kinds of PLGA hybrid scaffolds on 7 days. Data are presented as mean ± SD (n = 4). *p*‐values are calculated using one‐way ANOVA with Tukey's post hoc test. ^*^ *p* < 0.0332, ^**^ *p* < 0.0021, ^***^ *p* < 0.0002, ^****^ *p* < 0.0001, and “ns” indicates no significance.

In addition, the P80/D10/M10 and P80/D5/M15 groups exhibit a significantly greater number of migrated HUVECs than the other groups, and no significant difference was found between the P80/D10/M10 group and P80/D5/M15 group (**Figure** **5**A). Moreover, the tube formation assay showed that the HUVECs under the stimulation of P80/D10/M10 or P80/D5/M15 formed more capillary‐like network structures rather than the branching structures with discontinuous tubular walls observed in other groups. Quantitatively, the P80/D10/M10 group and P80/D5/M15 group has a significantly greater number of tubules than the other groups due to the stimulation of higher level of Mg ions (Figure 5B). Furthermore, to further confirm results obtained from the tube formation assay, the expression of HIF‐1α and VEGF proteins was also detected on day 7 using the western blot method. Our result showed that there were nearly 2.07‐ and 2.37‐times increases in HIF‐1α and VEGF expression in the P80/D10/M10 group to the CTL group, which were significantly higher than that of the other groups (Figure 5C). These results suggested that the controlled release of Mg ions from the incorporated MH‐NPs within PLGA hybrid scaffolds played a core part in promoting angiogenesis, and excessive increase of Mg ions by increasing incorporation of MH‐NPs into PLGA hybrid scaffolds showed no function on further enhancement of angiogenic activities. Moreover, P80/D10/M10 has the best cytocompatibility and angiogenic capacity.

### 2.6 In Vitro Modulation of Osteogenesis and Osteoclasis

At the late stage of scaffold implantation, the embedded DBM‐MPs in the PLGA hybrid scaffolds were gradually exposed along with the PLGA degradation, meanwhile, a small amount of Mg ions was slowly and stably released. These exposed DBM‐MPs and released Mg ions functioned together as a stimulator of osteogenesis as well as an inhibitor of osteoclasis, thus facilitating new bone integration to replace the implanted PLGA hybrid scaffold.

**Figure 6:**
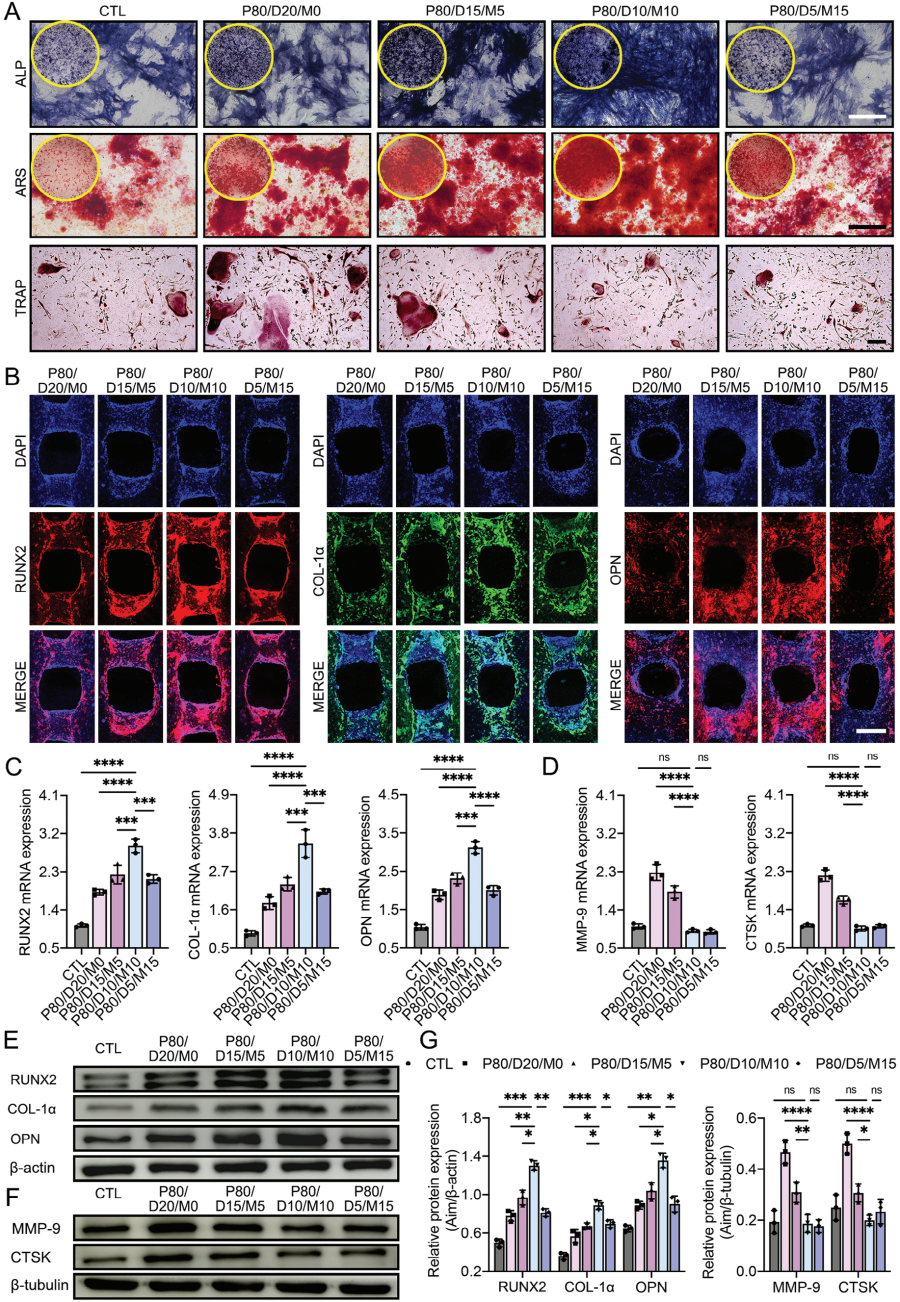
In vitro evaluation of the four PLGA hybrid scaffolds on osteogenesis and osteoclasis. A) ALP activity and calcium deposition were used to assess the osteogenic differentiation of BMSCs under scaffold stimulation. After 14 days of osteogenic induction, ALP staining was performed. At 21 days, ARS for calcium nodules. After 6 days of LPS and RANKL, osteoclast formation of RAW 264.7 cells was detected using TRAP staining. Scale bar = 250 µm. B) The osteogenic markers (RUNX2, COL‐1α, OPN) expressed in BMSCs cocultured with four kinds of PLGA hybrid scaffolds after 14 days of osteogenic induction. Scale bar = 300 µm. C,D) The osteogenic (RUNX2, COL‐1α, OPN) genes expressed in BMSCs and the osteoclast (MMP‐9, CTSK) genes expressed in RAW 264.7 cells under scaffold stimulation were evaluated using qRT‐PCR analysis. E–G) the levels of osteogenic protein (RUNX2, COL‐1α, OPN) markers expressed in BMSCs and the levels of the osteoclast protein (MMP‐9, CTSK) markers expressed in RAW 264.7 cells under 14 or 6 days of scaffold stimulation evaluated using western‐blot. Data are presented as mean ± SD (*n* = 3). *p*‐values are calculated using one‐way ANOVA with Tukey's post hoc test. ^*^ *p* < 0.0332, ^**^ *p* < 0.0021, ^***^ *p* < 0.0002, ^****^ *p* < 0.0001, and “ns” indicates no significance.

In this study, BMSCs were cocultured with the four kinds of PLGA hybrid scaffold, calcium deposition, alkaline phosphatase (ALP) activity, and expression of osteogenic markers are measured to comparatively evaluate their osteogenic inducibilities. First, ALP activity, as a pivotal indicator of initial osteogenic differentiation, and its expression in the BMSCs at days 7 and 14 were assessed by ALP staining (**Figure** **6**A; Figure S6, Supporting Information). As expected, the P80/D10/M10 group shows the highest ALP activity. Them, calcium nodules deposition, a crucial marker of late osteogenic differentiation, was visualized by Alizarin Red staining (ARS). After culturing for 21 days, calcium nodules appear in all groups, the P80/D10/M10 group has the most obvious and largest calcium nodules deposition (Figure 6A). Additionally, the osteogenic markers (RUNX2, COL‐1α, OPN) expressed in the BMSCs were stained by immunofluorescence staining, quantitative real‐time PCR (qRT‐PCR), and western blot. After culturing for 14 days, these osteogenic genes or markers (RUNX2, COL‐1α, OPN) in the P80/D10/M10 group showed the highest expression among all groups (Figure 6B,C,E,G).

Meanwhile, to verify the effects of scaffolds on osteoclast formation, RAW 264.7 cells were grown in culture dishes containing prefabricated P80/D20/M0, P80/D15/M5, P80/D10/M10, or P80/D5/M15 sheets and cultured in a complete medium containing LPS and nuclear factor 𝜅B receptor activator ligand (RANKL) for 6 days. Antitartrate acid phosphatase (TRAP) staining determined that the P80/D10/M10 and P80/D5/M15 groups showed similar osteoclast formation compared to the CTL group, and lower than the P80/D20/M0 and P80/D15/M5 groups (Figure 6A). We further examined the expression of osteoclast formation‐related genes and protein markers (MMP‐9 and CTSK) using qRT‐PCR together with western blot. The results showed that the expressions of MMP‐9 and CTSK in the CTL, P80/D10/M10, and P80/D5/M15 groups were similar without significant difference, but significantly lower than the P80/D20/M0 and P80/D15/M5 groups (Figure 6D,F,G). The above results showed that P80/D10/M10 has excellent osteogenic inducibility and can inhibit osteoclast formation in vitro.

### 2.7 Rat Cranial Bone Defect Repair by P80/D10/M10

**Figure 7:**
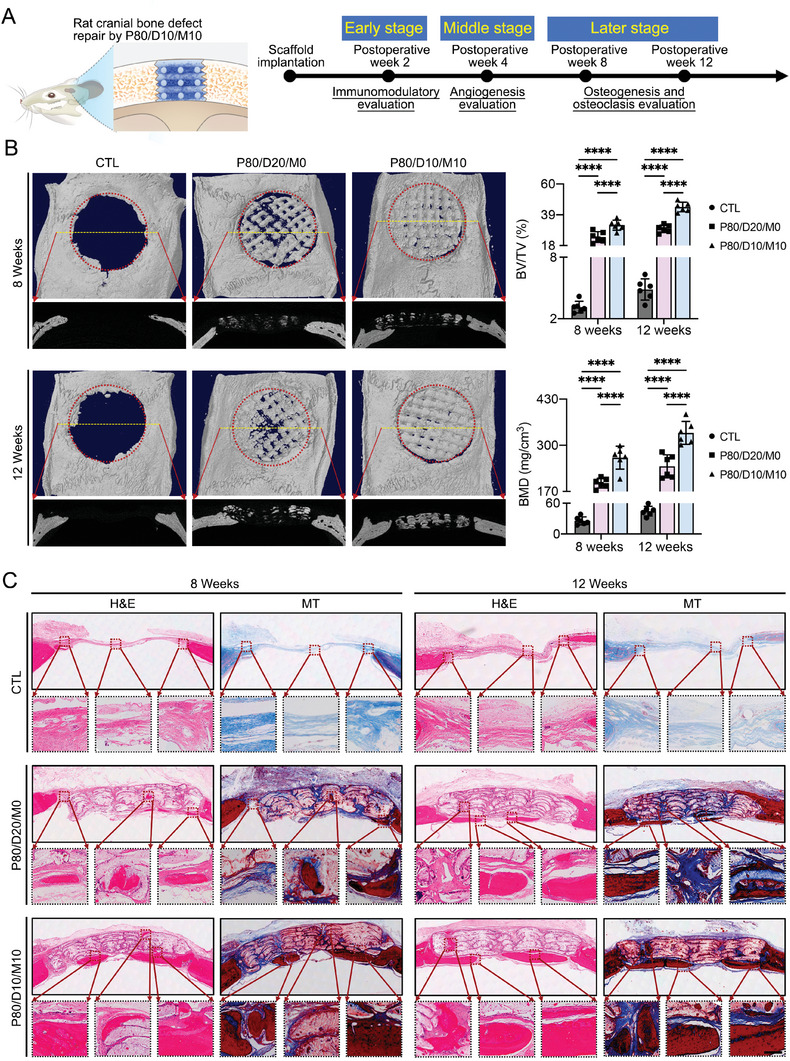
In vivo performance of the four PLGA hybrid scaffolds on bone defect repair. A) Schematic illustration showing the design and evaluation of scaffolds for the treatment of rat cranial defects. B) After 8 and 12 weeks of scaffold implantation, representative 3D‐reconstructed images, and 2D topography of the calvarial bone were obtained from micro‐CT scanning. BMD and BV/TV analysis obtained from micro‐CT results. C) H&E and Masson's trichrome (MT) stained sections of the implanted scaffold and newly‐formed bones at the skull defect site at 8 and 12 weeks. The image at the bottom is an enlarged view of the red frame. Scale bar = 150 µm. Data are presented as mean ± SD (*n* = 6). *p*‐values are calculated using one‐way ANOVA with Bonferroni post hoc test. ^*^ *p* < 0.0332, ^**^ *p* < 0.0021, ^***^ *p* < 0.0002, ^****^ *p* < 0.0001, and “ns” indicates no significance.

In view that the P80/D10/M10 shows the best properties for optimizing early immunomodulation, middle angiogenesis, and later osteogenesis in vitro, we just set three groups (CTL, P80/D20/M0, P80/D10/M10) in this study. This is more compliant with ethical requirements for laboratory animals. First, to confirm the therapeutic potential of P80/D10/M10 in bone repair, the P80/D10/M10 was implanted into a rat critical calvarial defect (8 mm in diameter) model, and endogenous bone regeneration was evaluated at postoperative week 8 and 12 by microcomputed tomography (micro‐CT) and histological staining (**Figure** **7**A).

As shown in the 2D tomography and 3D reconstruction images (Figure 7B), at postoperative week 8, owing to the absence of implants in the CTL group, there was almost no new bone formation within the defect site and only a very small amount of new bone formed near the severed ends. The P80/D10/M10 group had more new bone than the P80/D20/M0 group. After 12 weeks of implantation, more new bone is observed in the P80/D20/M0 and P80/D10/M10 groups compared with the CTL group, especially the P80/D10/M10 group. Quantitatively, the new bone in the P80/D10/M10 group showed significantly higher values in bone volume/tissue volume (BV/TV) and bone mineral density (BMD) than the CTL and P80/D20/M0 groups at both week 8 and week 12 after operation. Moreover, histological evaluation was used to further support the micro‐CT results and to verify the internal bone formation. The middle sagittal section of the entire bone defect area was stained with H&E and MT (Figure 7C). At postoperative week 8, the formation of new bone was not visible in the CTL group, but fibrous tissue containing a few new bones appeared in the pores of P80/D20/M0. The fibrous tissue and the new bone that appeared in the pores of P80/D10/M10 were more obvious and larger than the P80/D20/M0. At postoperative week 12, a small amount of new bone was also observed near the severed ends in the CTL group, while the pores of P80/D10/M10 showed more fibrous tissue infiltration and new bone formation compared to the P80/D20/M0. The residual scaffolds could be seen in some sections. In addition, the H&E‐stained images of animal viscera showed that the scaffolds were nontoxic to the organisms (Figure S7, Supporting Information).

**Figure 8:**
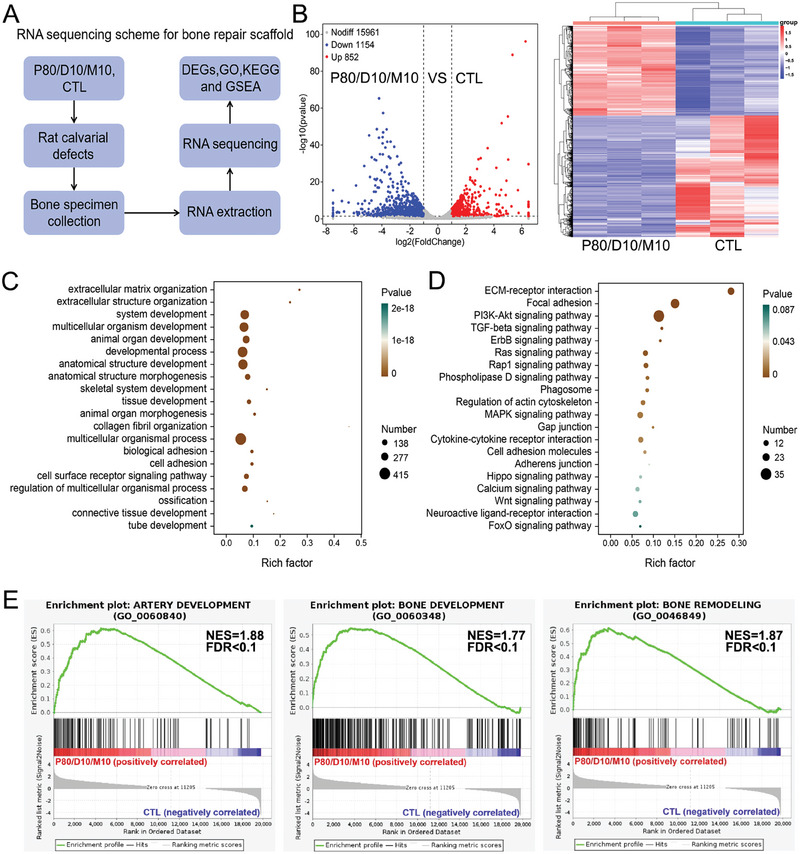
Transcriptome analysis of the scaffold's function to accelerate bone defect repair. A) RNA sequencing scheme for the implanted scaffold and the newly‐formed tissues. B) The DEGs in the P80/D10/M10 group compared with the CTL group. Heatmap analysis reveals the DEGs which are all up‐regulated or down‐regulated in the P80/D10/M10 group and CTL group. C,D) The top 20 up‐enrichment of GO terms (Category: Biological Process) and KEGG pathways (Category: Environmental Information Processing and Cellular Processes) in the P80/D10/M10 group compared with the CTL group. E) GSEA analysis of gene sets for artery development, bone development, and bone remodeling. NES, normalized enrichment score. FDR, false discovery rate. Three samples were measured per group.

In vivo experiments showed that the P80/D10/M10 had satisfactory abilities in bone formation. To further elucidate the underlying mechanism and related biological behaviors of P80/D10/M10‐induced bone regeneration, the tissue in rat calvarial bone defects with or without P80/D10/M10 implanted were collected at postoperative week 4 for transcriptomic analysis (**Figure** **8**A).

The analysis of differentially expressed genes (DEGs) revealed that the expression of 852 genes was up‐regulated and 1154 genes were down‐regulated in the P80/D10/M10 group (p‐value adjusted for multiple testing <0.05) compared to the CTL group (Figure 8B). To better illuminate the functions of these DEGs, the enrichment analysis for Gene Ontology (GO) terms was conducted for up‐regulated DEGs. These results suggest that the P80/D10/M10 promote the repair of rat critical calvarial defect likely through regulating the biological processes involved in “skeletal system development”, “Positive regulation of osteoblast differentiation”, “cell adhesion”, “cell surface receptor signaling pathway”, “connective tissue development”, “ossification” and “tube development” (Figure 8C). Kyoto Encyclopedia of Genes and Genome (KEGG) pathway enrichment analysis showed that these differentially expressed genes were enriched in some diabetic and immune‐related pathways, such as “PI3K‐AKT signaling pathway”, “TGF‐beta signaling pathway”, “MAPK signaling pathway”, and “Wnt signaling pathway” (Figure 8D). Consistently, we found that genes related to artery development, bone development, and bone remodeling were enriched significantly in the P80/D10/M10 by Gene Set Enrichment Analysis (GSEA) analysis (Figure 8E).

### 2.8 In Vivo Osteo‐Immunomodulation of P80/D10/M10

**Figure 9:**
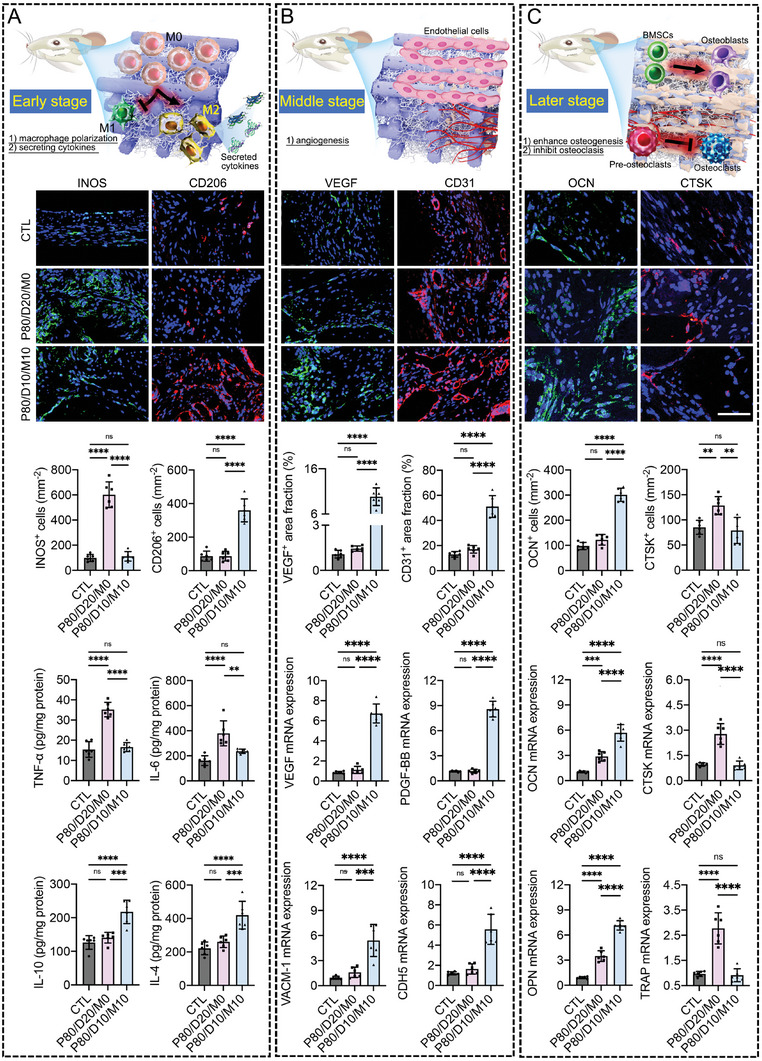
P80/D10/M10 shows the ability to offer an osteo‐friendly microenvironment in a stage‐matched pattern for enhanced bone regeneration. A) At postoperative week 2, the immunomodulatory function of scaffolds was evaluated by immunofluorescence staining for INOS and CD206 together with ELISAs for the TNF‐α, IL‐6, IL‐10, and IL‐4 in the newly‐formed tissue. B) At postoperative week 4, the function of scaffolds on stimulating neovascularization was evaluated by immunofluorescence staining for VEGF and CD31, and qRT‐PCR for angiogenesis‐related genes (VEGF, PDGF‐BB, VACM‐1, CDH5) in the fibrous tissues. C) At postoperative week 8, the osteogenesis and osteoclasis within different implanted scaffolds were evaluated by detecting the expressions of osteogenesis markers (OCN, OPN) and osteoclasis (CTSK, MMP‐9) markers. Scale bar = 50 µm. Data are presented as mean ± SD (*n* = 6). *p*‐values are calculated using one‐way ANOVA with Bonferroni post hoc test. ^*^ *p* < 0.0332, ^**^ *p* < 0.0021, ^***^ *p* < 0.0002, ^****^ *p* < 0.0001, and “ns” indicate no significance.

At the early stage of implantation, inflammatory cells infiltrate into the porous structure of the 3D‐printed P80/D20/M0 or P80/D10/M10 scaffold, which was eventually filled and wrapped by newly‐formed fibrous tissues due to the rejection reaction of the organism.^[^ ^28^ ^]^ After 2 weeks of implantation, the implanted scaffolds and infiltrated fibrous tissues were collected to evaluate the immunomodulatory effects of the P80/D10/M10 scaffold by immunofluorescence staining and Enzyme‐Linked Immunosorbent Assays (ELISAs). Immunofluorescence staining of the INOS (a marker of M1 phenotype macrophages) and CD206 (a marker of M2 phenotype macrophages) is selected to characterize the effect of different implants on macrophage polarization. As shown in **Figure** **9**A, the proportion of M1 macrophages in the P80/D20/M0 group is the highest, while the P80/D10/M10 group showed the highest proportion of M2 macrophages in the newly‐formed fibrous tissues. In addition, the P80/D10/M10 group also showed significantly lower levels of TNF‐α and IL‐6, as well as higher levels of IL‐10 and IL‐4 than other groups. These results confirm that P80/D10/M10 can optimize the inflammatory response at the initial stage of implantation.

### 2.9 In Vivo Neovascularization Stimulated by P80/D10/M10

Following the early stage of inflammation, neovascularization gradually formed within the infiltrated fibrous tissues, as characterized by the densest blood vessel branches. At 4 weeks after scaffold implantation, the implanted scaffolds and infiltrated fibrous tissues were harvested to evaluate the angiogenesis within the scaffold. As shown in Figure 9B, immunofluorescence images showed that the infiltrated fibrous tissues in the P80/D10/M10 group showed significantly more VEGF expression and CD31‐positive vessel branches than the other groups. Besides, angiogenesis‐related genes (VEGF, PDGF‐BB, VACM‐1, CDH5) in the fibrous tissues of the P80/D10/M10 group expressed significantly higher than the fibrous tissues of other groups. These results showed that the P80/D10/M10 can promote neovascularization at the middle stage of implantation.

### 2.10 In Vivo Osteogenesis and Osteoclasis Influenced by P80/D10/M10

At the later stage of implantation, to clarify the osteogenesis and osteoclasis within the implanted scaffold, we applied immunohistochemical staining to detect the expression levels of osteogenic (OCN) or osteoclastic (CTSK) protein in the fibrous tissue and new bone at postoperative week 8 (Figure 9C). Besides, the expression of osteogenic‐related genes (OPN and OCN) or osteoclastic‐related genes (CTSK and TRAP) in the fibrous tissue and new bone were quantitatively examined using qRT‐PCR. Immunohistochemical staining showed that OCN was significantly expressed in the P80/D10/M10 group to the CTL and P80/D20/M0 groups, while more CTSK‐positive cells located within the fibrous tissue and new bone from the P80/D20/M0 group in comparison with the CTL and P80/D10/M10 groups. qRT‐PCR result quantitatively determined that the fibrous tissue and new bone in the P80/D10/M10 group expressed significantly higher levels of OPN and OCN genes than the other group. while osteoclastic‐related genes (TRAP and CTSK) in the P80/D10/M10 group were significantly lower than the P80/D20/M0 group. Taken together, these results indicated that the P80/D10/M10 notably enhanced osteogenesis and inhibited osteoclasis.

## 3 Discussion

Natural bone tissue has a high self‐healing capacity to realize regeneration as cracks, simple‐type fractures, or minor bone defects happen.^[^ ^1^, ^29^ ^]^ But the bone defects are larger than the critical size, it is unable to achieve self‐regeneration. Currently, plenty of engineered bone scaffolds with excellent osteoinductive activity have been developed for repairing critical‐size bone defects, but their compositional materials are far from ideal owing to their degradation productions without the capabilities of successively building an osteo‐friendly microenvironment for bone defect repair. Herein, we successfully fabricated a 3D‐printed PLGA hybrid scaffold containing biomimetic DBM‐MPs and multifunctional MH‐NPs with an optimized proportion. This 3D‐printed PLGA/DBM‐MPs/MH‐NPs hybrid scaffold (P80/D10/M10) showed similar mechanical properties to normal trabecular bone, suitable as a bone substitute for filling bone defects. More importantly, as its degradation, the gradually released Mg ions and exposed biomimetic DBM‐MPs could sequentially modulate early inflammation, middle vascularization, later osteogenesis, and osteoclasis, thus providing a stage‐matched osteo‐friendly microenvironment for bone defect repair. This P80/D10/M10 scaffold may be a promising bone substitute for human patients with critical‐size bone defects in the future.

PLGA, due to its simple processing by 3D printing, biocompatibility, and biodegradability, is one of the most widely used biomedical polymers in bone tissue engineering.^[^ ^5^, ^6^ ^]^ Meanwhile, DBM‐MPs, owing to their biocompatible, biomimetic, and osteogenic properties,^[^ ^11^ ^]^ are an ideal additive of PLGA to improve its osteogenic properties. Moreover, MH‐NPs, as a type of biocompatible and multifunctional magnesium ceramic particle, are also suitable for blending into PLGA as a neutralizer of the acidic hydrolysate during PLGA degradation and a modulator of osteo‐microenvironment.^[^ ^16^, ^20^ ^]^ Considering that the DBM‐MPs contain lots of osteogenic cytokines/growth factors,^[^ ^10^ ^]^ to ensure the bioactivities of these inherent osteogenic cytokines/growth factors, a novel LT‐RP 3D‐printing technology was used for fabricating the PLGA/DBM‐MPs/MH‐NPs hybrid scaffolds. Compared to the conventional scaffold preparation techniques, the 3D printing technique can accurately produce bone substitute matching bone defects in individual patients.^[^ ^3c^ ^]^ Specifically, 3D printing based on computational topology design has made it possible to fabricate bone scaffolds with controlled porous structures, which play a vital role in bone formation by facilitating endogenous cell migration, vascular, as well as metabolites, and nutrient exchange.^[^ ^30^ ^]^ Previous studies indicated that the pore sizes of 3D‐printed PLGA hybrid scaffold ranged from 400–500 µm, showing a similar geometry to trabecular bone and having good biocompatibility, biodegradability, and osteoconductivity in vitro and in vivo.^[^ ^16^, ^31^ ^]^ In this study, these PLGA/DBM‐MPs/MH‐NPs hybrid scaffolds were also produced with pore sizes of 500 µm using LT‐RP 3D‐printing technology, which could endow the interconnectivity of scaffold macropores and micropores, thus allowing migrated cells to adhere, proliferate, and differentiate into osteocytes as well as form bone extracellular matrix on the scaffolds.

As a bone substitute to repair bone defects, 3D‐printed bone scaffolds first require excellent biocompatibility and similar mechanical properties to the trabecular bone of the insertion site.^[^ ^32^ ^]^ Given the inferior mechanical properties and tiresome acidic degradations of PLGA, DBM‐MPs were novelty fabricated and integrated into PLGA to improve their mechanical properties in this study. However, Young's modulus of P80/D20/M0 was ≈41.1 ± 2.2 MPa, which was significantly lower than that of trabecular bone (75–184 MPa),^[^ ^33^ ^]^ and cannot meet initial mechanical properties for implantation. In general, the mechanical property of the porous scaffold, particularly the flexural modulus, is determined not only by the intrinsic modulus of the matrix and additive but also by extrinsic properties, such as structure and particle distribution.^[^ ^34^ ^]^ According to SEM images, the DBM‐MPs showed a heterogeneous particle size, leading to uneven distribution in the PLGA, which causes a reduction in interfacial force between PLGA and DBM‐MPs. Moreover, the DBM‐MPs showed limited ability to neutralize the acidic degradation product of PLGA. As a multifunctional additive in bone tissue engineering, MH‐NPs were confirmed to have the ability to enhance the mechanical properties of PLGA hybrid scaffolds and efficiently neutralize the acidic degradation product of PLGA. More importantly, MH‐NPs can be blended into PLGA forming air bubbles, which is more convenient for cell adhesion and migration as well as oxygen and nutrients exchange. Therefore, decreasing DBM‐MPs and adding MH‐NPs into PLGA is a promising strategy to improve polymers' mechanical properties and neutralize acidic degradation. As shown in our physical analysis, the three MH‐incorporated PLGA/DBM‐MPs hybrid scaffolds presented a similar microstructure to the P80/D20/M0 scaffold, but their mechanical strengths were improved. Especially, the mechanical properties of the P80/D10/M10 or the P80/D5/M15 reached the level of natural trabecular bone, indicating that the two scaffolds can provide initial mechanical support for bone defect repair at the beginning. As we expected, the pH value of the in vitro degradation solution from the P80/D10/M10 was neutral or slightly alkaline, while the degradation solution from the other scaffold presented an acidic or alkaline state. In vitro cell studies also demonstrated that the PLGA scaffold integrated 10% DBM‐MPs and 10% MH‐NPs were suitable for cell adhesion and proliferation.

Besides the initial mechanical strength and good biocompatibility, our study also determined that the incorporated biomimetic DBM‐MPs and multifunctional MH‐NPs make the P80/D10/M10 capable of stage‐matched modulating early inflammation, middle vascularization, and late osteogenesis during bone defect repair. The early inflammatory phase is initiated after bone injury, and prolonged or magnified pro‐inflammatory reaction at this phase leads to poor bone regeneration.^[^ ^26c^ ^]^ The manipulation of macrophage phenotype switching from pro‐inflammatory M1 phenotypes to pro‐regenerative M2 phenotypes appears to be a promising trigger that weakens early inflammatory reaction.^[^ ^13a^ ^]^ Thus, various kinds of bone scaffolds were designed to enhance bone regeneration by facilitating sequential M1 and M2 macrophage polarization in the early inflammatory stage.^[^ ^35^ ^]^ Recently, some studies have determined that Mg ions were able to modulate the sequential activation of M1 and M2 phenotypes in macrophages.^[^ ^17^, ^36^ ^]^ As shown in our study, the P80/D10/M10 polarized more RAW264.7 cells toward the M2 phenotype and released anti‐inflammatory cytokines than the other scaffolds. Besides, in rat calvarial defect implanted with P80/D10/M10, early inflammation was alleviated, characterized by a gradual increase in M2 macrophages, rapid decline in M1 macrophages, and decreasing expression of pro‐inflammatory factors. Therefore, we speculate that the blended MH‐NPs in the P80/D10/M10 gradually dissolved to produce Mg ions, which could build a pro‐regenerative immune microenvironment by inducing an anti‐inflammatory phenotype switch of macrophages and decreasing inflammatory factors. In the middle fibrovascular phase, the neovascularization network gradually forms in the bone defect site for a continuous supply of oxygen, nutrients, and functional cells to support the following bone formation and remodeling. Currently, there have been many attempts at the addition of angiogenic substances into bone substitutes to stimulate bone repair.^[^ ^37^ ^]^ Similarly, in our study, a burst of neovascularization was observed in the P80/D10/M10‐treated animals on week 4, indicating that the released Mg ions from the P80/D10/M10 have the function of enhancing neovascularization in the bone defect site. When the middle fibrovascular phase is completed, the bone defect repair is processed into the late bone formation and remodeling phase. In this phase, the recruited MSCs from the bone marrow, periosteum, adjacent soft tissues, and peripheral circulation are stimulated by the osteo‐environments around P80/D10/M10 to form a soft callus, then mineralized into a hard callus with irregular woven bone, finally remodeled into the lamellar bone through an orderly bone resorption and formation process.^[^ ^12^, ^38^ ^]^ Thus, part of tissue‐engineered bone scaffolds were developed by loading osteogenic molecules to stimulate bone formation and remodeling.^[^ ^39^ ^]^ In consideration that Mg ions can promote osteogenesis through PI3K‐Akt and Wnt signaling pathway,^[^ ^16d^ ^]^ we believe that these released Mg ions from the P80/D10/M10 are the first factor that stimulates new bone formation in the defect site. In addition, a previous study determined that DBM shows superior osteogenic properties owing to inherent osteogenic substances.^[^ ^11^ ^]^ With the degradation of P80/D10/M10, the incorporated DBM‐MPs were gradually exposed to the migrated stem cells, which also functioned as an enhancer for new bone formation. According to the in vivo transcriptome analysis, the newly‐formed tissues in the P80/D10/M10 group showed gene enrichment in artery development, bone development, and bone remodeling, which could serve as evidence to support our speculations. Next step, some experiments of molecular biology should be designed to deeply elucidate the mechanisms of P80/D10/M10‐stimulated bone formation.

## 4 Conclusion

In this study, a novel PLGA hybrid bone scaffold (P80/D10/M10) was developed by LT‐RP 3D‐printing technology, in which biomimetic DBM‐MPs and multifunctional MH‐NPs were creatively incorporated into the PLGA with an optimized proportion as print ink. In vitro, results confirmed that this P80/D10/M10 showed excellent properties in mechanics, biocompatibility, and biodegradability and, at the same time, presented superior stimulations in osteo‐immunomodulation, angiogenesis, and osteogenesis. In vivo, results indicated that this P80/D10/M10 can offer an osteo‐friendly microenvironment in a stage‐matched pattern for the enhancement of endogenous bone regeneration, namely, optimization of early inflammation, middle neovascularization, and later bone formation. Transcriptomic analysis suggested that the in vivo performance of the P80/D10/M10 scaffold on bone defect repair was mostly attributed to regulating artery development, bone development, and bone remodeling. Therefore, the P80/D10/M10 scaffold is an excellent bone substitute that can be used in the treatment of bone defects with stage‐matched modulation of inflammation, vascularization, and osteogenesis.

## 5 Experimental Section

### Materials

PLGA (Medical grade, LA: GA = 75:25) was purchased by Regenovo Biotechnology Co., Ltd., Hangzhou, China. 1,4‐Dioxane was obtained in Aladdin Biochemical Technology Co., Ltd., Shanghai, China. DBM‐MPs were autonomously synthesized by the laboratory. MH‐NPs with an average particle size of 50 nm were purchased from Zhongke Leiming Technology Co., Ltd., Beijing, China. Fetal bovine serum (FBS), *α*‐MEM, DMEM, penicillin–streptomycin, and trypsin were purchased from Gibco Life Technologies Co. Grand Island, USA. Dexamethasone, ascorbic acid, and *β*‐Sodium Glycerol 3‐phosphate were purchased from Solarbio Science&Technology Co., Ltd., Beijing, China. PBS, Tween‐20, and bovine serum albumin (BSA) were bought from Sigma–Aldrich Products (USA).

### Preparation of the DBM‐MPs

Trabecular bone tissues were dissected from the pig's spinal vertebrae at a local slaughterhouse and trimmed into small blocks. After wrapping the bone blocks with gauze, they were immersed into a 4 °C 2% SDS (Aladdin, China) solution containing 0.1% Triton X‐100 (Beyotime, China) for 12 h under vigorous agitation. Then the bone blocks were washed with PBS at 4 °C three times (8 h per each time) and digested in a nuclease solution (containing 500 U mL^−1^ DNase Type I and 1 mg mL^−1^ RNase) with agitation at 37 °C for 12 h. After washing with PBS three times (8 h per each time) and lyophilizing in a vacuum freeze‐drier (FD8‐5T, SIM, Newark, NJ), the DBM was acquired. After that, the DBM blocks were ground into DBM powders using a low‐temperature grinder machine (Servicebio, Wuhan, China), and then sifted through steel wire sieves with an aperture of 74 µm to get DBM‐MPs.

### Evaluation of DBM‐MPs and MH‐NPs

Natural bone tissue (NBT) was selected as the control. DBM and NBT were fixed with 4% Paraformaldehyde for 48 h, then decalcified and embedded within paraffin. H&E staining, DAPI staining, and PicoGreen DNA assay kit (Invitrogen, USA) were used to observe the clearance of cellular components. SR staining was used to evaluate collagen retention in the DBM. At the same time, the DBM and NBT fixed with 2.5% Glutaraldehyde at 4 °C overnight were analyzed by SEM (Mira4 LMH, TESCAN, Czech Republic) to observe the microscopic changes of the bone matrix before and after decellularization. Meanwhile, the content of calcium (Ca) and phosphorus (P) were evaluated by EDS analysis. In addition, the particle size of DBM‐MPs was evaluated with a laser particle sizer (Mastersizer 2000, UK). The microstructure of MH‐NPs was observed by TEM (JEM‐F200, Japan) and the crystal phase of MH‐NPs was evaluated by XRD (Rigaku SmartLab SE, Japan).

### Fabrication of PLGA/DBM‐MPs/MH‐NPs Scaffolds by LT‐RP 3D‐Printing Technology

Considering that various PLGA‐based bone scaffolds contained ≈70–90% PLGA, the study also selected 80% as the fabrication proportion to prepare the scaffold.^[^ ^40^ ^]^ In order to optimize the incorporation proportion of DBM‐MPs and MH‐NPs in PLGA hybrid scaffold, four kinds of 3D‐print inks were prepared, namely P80/D20/M0: 80% PLGA incorporated 20% DBM‐MPs; P80/D15/M5: 80% PLGA incorporated 15% DBM‐MPs and 5% MH‐NPs; P80/D10/M10: 80% PLGA incorporated 10% DBM‐MPs and 10% MH‐NPs; P80/D5/M15: 80% PLGA incorporated 5% DBM‐MPs and 15% MH‐NPs. At room temperature, PLGA was dissolved in 1, 4‐dioxane, and stirred magnetically for 30 min. Then, DBM‐MPs and MH‐NPs were added into the dissolved PLGA according to the above‐mentioned ratio and stirred for 10 min to form a homogenized suspension. Using an ultra‐low temperature platform, four kinds of PLGA hybrid scaffolds (P80/D20/M0, P80/D15/M5, P80/D10/M10, or P80/D5/M15) were printed with a shape of circular body (8 mm in diameter and 1.5 mm in height), using a pore diameter of 500 µm and a filament diameter of 300 µm as the target parameters. And then, these 3D‐printed scaffolds were lyophilized in a vacuum freeze‐drier (FD8‐5T, SIM, Newark, NJ).

### Evaluation of PLGA/DBM‐MPs/MH‐NPs Scaffolds

SEM was used to observe the macropores and microscopic characteristics of the four groups of scaffolds. Meanwhile, the distribution of carbon (C), calcium (Ca), and magnesium (Mg) in the scaffolds was analyzed by EDS (Mira3 LMH, Tescan, Czech Republic). The porosity of each scaffold was calculated using the anhydrous ethanol replacement method with the following formula: Porosity (%) = (V1‐V3)/(V2‐V3) × 100%, where *V1* was the volume of anhydrous ethanol added, *V2* was the volume after soaking the scaffold in anhydrous ethanol and extracting vacuum, and *V3* was the remaining volume of anhydrous ethanol after scaffold removal. The scaffolds with a diameter of 8 mm and a height of 10 mm were used for mechanical testing using a mechanical universal testing machine (INSTRON 5982, USA) with a compression speed of 1 mm min^−1^ to obtain stress–distance curves. Young's modulus of scaffolds was calculated from the acquired stress‐distance curves. In order to evaluate the hydrophilicity of the four scaffolds, a contact angle measuring instrument (Lauda Scientific LSA100, Germany) was used to measure water droplet angle data. Besides, the degradation behavior of the four scaffolds was tested as follows: scaffolds were immersed in PBS with a mass volume ratio of 0.1 g mL^−1^ for 84 days at 37 °C and 100 rpm. At the planned timeline, the degradation solutions were collected, while the immersed scaffolds were lyophilized in a vacuum freeze‐drier (FD8‐5T, SIM, Newark, NJ). After weighing the mass of those lyophilized scaffolds, they were immersed in an equal volume of fresh PBS again. A pH meter (STARTER 3100, OHAUS, USA) was used to measure the pH value of these collected degradation solutions. An inductively coupled plasma optical emission spectrometry (ICP‐OES, SPECTROBLUE, Germany) was also used to measure the concentration of Ca and Mg ions in these collected degradation solutions.

In order to evaluate the biocompatibility of the four kinds of scaffolds, ≈2.5 × 10^4^ rat BMSCs (passage 3) were seeded on the scaffold, and a Live/Dead Cell kit (BestBio, China) was used to detect the cell viability on day 4. Meanwhile, the BMSCs adhesion on the scaffold was evaluated by staining iFluor 647‐labeled phalloidin (Yeasen, China) together with DAPI (Solarbio, China) on days 1, 4, and 7. In addition, the CCK‐8 assay (Beyotime, China) was used to evaluate the BMSCs proliferation on the scaffolds on days 1, 4, and 7.

### In Vitro Immunomodulation, Angiogenesis, Osteogenesis, and Osteoclasis of PLGA/DBM‐MPs/MH‐NPs Scaffolds

*Immunocytochemistry*: The immunomodulatory properties of the scaffold were evaluated using a co‐culture system of RAW 264.7 cells and scaffolds. Briefly, RAW 264.7 cells were directly implanted on the scaffold at a density of 5 × 10^4^ per well, and cultured within a 24‐well plate. The culture medium containing LPS (Solarbio, China) at a concentration of 1 µg mL^−1^ was set up as an LPS group. After 3 days of co‐culture, the RAW 264.7 cells were fixed with 4% paraformaldehyde for 15 min, permeabilized with 0.1% Triton X‐100, and sealed with goat serum at room temperature for 45 min, followed by overnight incubation with a specific primary antibody. The next day, these cells were labeled with fluorescent secondary antibody, PBS washed, and retained with DAPI before the observation with a fluorescence confocal microscope.

The osteogenesis properties of each scaffold were evaluated using a co‐culture system of rat BMSCs and scaffolds. Briefly, after the scaffolds were placed at the bottom of a 24‐well plate and soaked with culture medium overnight, rat BMSCs were directly seeded on the scaffold at a density of 5 × 10^4^ per well. Subsequently, the rat BMSCs were cultured for 14 days within an osteogenic induction medium, and osteogenic marker proteins were visualized by immunofluorescence staining. The relevant antibodies used are as follows: INOS (ab178945, Abcam, 1:250), CD86 (13395‐1‐AP, Proteintech, 1:100), CD163 (ab182422, Abcam, 1:200), CD206 (#24 595, Cell Signaling Technology, 1:400), Runx‐2 (20700‐1‐AP, Proteintech, 1:100), Col‐1α (66761‐1‐lg, Proteintech, 1:100), OPN (22952‐1‐AP, Proteintech, 1:100).

*ELISAs*: Using a co‐culture system of RAW 264.7 cells and scaffolds, RAW264.7 cells were seeded on the scaffold at a density of 5 × 10^4^ per well and cultured on a 24‐well plate. Meanwhile, the blank group and the LPS group were set up. On day 3 of cultivation, the cell supernatant was collected for measuring TNF‐α, IL‐6, IL‐1β, IL‐10, IL‐4, VEGF‐α, and BMP‐2 content according to the instructions of the ELISAs kit (Cusabio or Elabscience, China).

*qRT‐PCR*: qRT‐PCR was used to detect the expression of INOS, CD86, CD163, CD206, Runx2, Col‐1α, OPN, MMP‐9, and CTSK. Briefly, the collected RAW 264.7 cells or rat BMSCs were used to extract total RNA with TRIzol reagent (CW Biotech, China), and cDNA was synthesized using a reverse transcription kit (Takara, Japan). cDNA was then amplified by qPCR system, all in 20 µL of the final reaction mixture. GAPDH was used as a reference for data processing. The relevant primer sequences are shown in Table S1 (Supporting Information).

*Transwell Assay*: The chemotactic effect of the scaffold on HUVECs was assayed by transwell migration assay. Briefly, the scaffolds were placed in the lower chamber with a culture medium for 1 week. Then, 3 × 10^4^ HUVECs were resuspended with FBS‐free medium and added to the upper chamber of a 24‐well transwell chamber (Corning, USA). After 24 h, the chambers were placed in 4% paraformaldehyde and fixed for 20 min, and cotton swabs were used to wipe the cells from the upper chamber. The migrated cells were stained with 0.01% crystal violet (W/V) for 10 min and observed under an inverted microscope for counting.

*Tube Formation*: Matrigel with 100 uL (BD Bioscience, USA) was added to the bottom of pre‐cooled 24‐well plates and then solidified at 37 °C for 30 min. The scaffolds were placed in the upper chamber and immersed in a culture medium for 7 days. HUVECs were seeded to the lower chamber of a 24‐well transwell chamber (Corning, USA) at a density of 8 × 10^4^ per well. After 6 h of incubation, the cells were stained with Calcein‐AM, and the number and diameter of lattice‐like shapes were counted under fluorescence microscopy (DMi8, Leica, Germany).

*Western‐Blot*: Total proteins from HUVECs, rat BMSCs, and RAW264.7 cells were extracted using TPEB buffer (Invitrogen, USA), and sample concentrations were assessed using the BCA method (Beyotime, China). The samples were then subjected to SDS electrophoresis and transferred to PVDF (Millipore, USA). Primary antibodies were incubated after 1 h of skim milk closure: VEGF (19003‐1‐AP, Proteintech, 1:2000), HIF‐1α (ab179483, Abcam, 1:1000), Runx‐2 (20700‐1‐AP, Proteintech, 1:1000), Col‐1α (66761‐1‐lg, Proteintech, 1:5000), OPN (22952‐1‐AP, Proteintech, 1:4000), MMP‐9 (10375‐2‐AP, Proteintech, 1:2000), CTSK (ab187647, Abcam. 1:5000). Overnight on a shaker at 4 °C and three washes with TBST, the samples were incubated with enzyme‐linked secondary antibodies (HS101‐01/HS201‐01, TransGen, 1:5000) for 1 h. Protein signals were visualized using a chemiluminescence imaging instrument (Amersham ImageQuant 800, China), and then quantitatively analyzed using Image‐J software.

*ALP and ARS Staining*: ALP staining or ARS staining was used to assess the osteogenic differentiation of rat BMSCs cultured on scaffolds. After 24 h of culture in 24‐well plates within an osteogenic medium, the rat BMSCs were sequentially fixed with paraformaldehyde at 7, 14, or 21 days, and then stained with ALP chromogenic kit (Beyotime, China) or ARS solution (Starfish Biotechnology, China).

*TRAP Staining*: RAW 264.7 cells were seeded on the scaffold at a density of 2 × 10^4^/well and cultured in a 24‐well plate within a medium containing 100 ng mL^−1^ RANKL (Novoprotein, China) and 1 µg mL^−1^ LPS. After 6 days of culture, a TRAP assay kit (Servicebio, China) was performed to observe the osteoclasis of RAW 264.7 cells.

### In Vivo Experiments

*Bone defect model and scaffold implantation*: Animal experiments of this study were approved by the Experimental Animal Ethics Committee of Xiangya Hospital, Central South University (Grant No. 202 103 171). The in vivo performance of P80/D10/M10 on bone defect repair was evaluated using a rat cranial bone defect model. Briefly, 8‐week‐old male Sprague Dawley (SD) rats were purchased from Hunan Silaikejingda Experimental Animal Co., Ltd., China. After the rat was anesthetized with 2.5% Pentobarbital sodium (40 mg k^−1^g), a surgical incision was performed along the median line of the rat's skull to expose the cranial bone. A full‐thickness bone defect with a diameter of 8 mm was created at the center of the sagittal suture of the rat skull. In the P80/D10/M10 group, the P80/D10/M10 was implanted into the bone defect site. In the P80/D20/M0 group, the P80/D20/M0 was implanted into the bone defect site, while the rats in the CTL group were injected with an equal volume of saline at the bone defect site. Finally, the incision was closed layer by layer, and antibiotics were administered for 3 consecutive days after surgery.

*Micro‐CT evaluation*: At postoperative weeks 8 and 12, the whole skull with bone defects was dissected and fixed with 4% paraformaldehyde. A micro‐CT machine (SCANCO Medical AG, Bruttisellen, Switzerland) was used to scan the whole skull. After scanning, using CTAn, DataViewer, and CTVol software, the 3D reconstruction of the skull was visualized. Meanwhile, BV/TV and BMD were calculated.

*Histological analysis*: The fixed skulls were decalcified with 10% EDTA solution, then dehydrated and embedded within paraffin. The specimens acquired at postoperative weeks 8 and 12 were cut midsagittally through the bone defect site and stained with hematoxylin and eosin and Masson trichrome for descriptive histological evaluation of new bone. In addition, the specimens acquired at postoperative weeks 2, 4, and 8 were sectioned, and stained with immunofluorescence staining for in vivo evaluation of immunomodulation, angiogenesis, osteogenesis, and osteoclasis. The relevant antibodies used are as follows: INOS (ab178945, Abcam, 1:250), CD206 (#24 595, Cell Signaling Technology, 1:400), VEGF (19003‐1‐AP, Proteintech, 1:100), CD31 (GB11063‐2‐100, Servicebio, 1:200), OCN (23418‐1‐AP, Proteintech, 1:200), and CTSK (11239‐1‐AP, Proteintech, 1:100). The immunofluorescence‐stained images of the target proteins were quantified using Image‐J software.

*RNA sequencing and analysis*: At postoperative week 4, the implanted scaffold and the attached tissues acquired from the skull defect site were dissected for transcriptome analysis. Briefly, total RNA was extracted with Trizol reagent and delivered to Personalbio Biotech (Shanghai, China) to create 6 sets of sample libraries (3 sets of P80/D10/M10 and 3 sets of CTL). Sequencing libraries were created using the NEBNext Ultra II RNA Library Prep Kit and sequenced on the illumina novaseqPE150 platform. Next, indexing of the reference genome was constructed using HISAT2 (v2.1.0), raw expression of genes was counted using HTSeq (v0.9.1), and then normalized expression volume using FPKM. Differential expression analysis was performed using DESeq (v1.38.3) software. For biological functions, the GO, KEGG pathway enrichment analysis, and GSEA dataset analysis were performed sequentially.

*ELISAs*: At postoperative week 2, the implanted scaffold and the attached tissues acquired from the skull defect site were dissected for ELISAs. Briefly, the specimen was immediately frozen in liquid nitrogen after dissection, and then crushed into a fine powder using a precooled Bio‐Pulverizer (BioSpecs, Bartlesville, OK, USA). The powdered tissue was extracted and centrifuged to collect the clear tissue extract. Protein concentration in the extract was determined using a Pierce BCA protein assay kit (Thermo Fisher Scientific Inc, Rockford, IL, USA). After that, Rat ELISAs kits for TNF‐α, IL‐6, IL‐10, and IL‐4 (Cusabio or Elabscience, China) were used to measure protein levels. Concentrations of TNF‐α, IL‐6, IL‐10, and IL‐4 were normalized to total protein concentrations determined using the Pierce BCA protein assay.

*qRT‐PCR*: At postoperative weeks 4 and 8, the implanted scaffold and the attached tissues acquired from the skull defect site were dissected, and qRT‐PCR was used to detect the expression of VEGF, PDGF‐BB, VACM‐1, CDH5, OCN, OPN, TRAP, and CTSK. Briefly, the dissected tissues from the bone defect site were retrieved, and homogenized, and total RNA was extracted with TRIzol reagent (CW Biotech, China), and cDNA was synthesized using a reverse transcription kit (Takara, Japan). cDNA was then amplified by qPCR system, all in 20 µL of the final reaction mixture. GAPDH was used as a reference for data processing. The relevant primer sequences are shown in Table S1 (Supporting Information).

### Statistical Analysis

All quantitative data were expressed as mean ± standard deviation, and the data were statistically analyzed using GraphPad Prism, SPSS25 software. For the in vitro experiments, it was repeated at least three times. An unpaired *t*‐test was used for the comparison between the two groups, while one‐way ANOVA with Tukey's post hoc test was used for the comparison above two groups. As for the in vivo experiments, the results were compared using a one‐way ANOVA with Bonferroni post hoc test. ^*^ indicated *p* < 0.0332, which was considered a statistically significant difference, while ^**^ indicates *p* < 0.0021, ^***^ indicates *p* < 0.0002, ^****^ indicates *p* < 0.0001.

## Conflict of Interest

The authors declare no conflict of interest.

## Author Contributions

C.C., and Y.Z. contributed to the conception of this study and supervised the experiments. C.C. and Y.Y. wrote and revised the manuscript. Y.Y. and Y.X. fabricated the DBM‐MPs and printed the 3D scaffolds. Y.Y. and M.O. preformed the cytology expriments. M.O., S.Z., and H.L. preformed the surgery of animal models . Y.Z., L.C., Y.M., and B.S. conducted Micro‐CT analysis. Y.Y., M.Z. and B.L. evaluated to the characterizations of the 3D scaffolds. Y.Z., Z.L., H.L., and R.Z. discussed the experimental design of this study.

## Supporting information

Supporting Information

## Data Availability

The data that support the findings of this study are available from the corresponding author upon reasonable request.
