# Supplementary material for: Three Birds, One Stone: An Osteo‐Microenvironment Stage‐Regulative Scaffold for Bone Defect Repair through Modulating Early Osteo‐Immunomodulation, Middle Neovascularization, and Later Osteogenesis
Source: Adv Sci (Weinh). 2023 Dec 7;11(6):2306428. doi: 10.1002/advs.202306428 (PMC10853759; doi:10.1002/advs.202306428)
Supplement: Supplementary file 1 — Supporting Information [file ADVS-11-2306428-s001.pdf]

ADVANCED SCIENCE
Open Access

Supporting Information

for *Adv. Sci.*, DOI 10.1002/advs.202306428

Three Birds, One Stone: An Osteo-Microenvironment Stage-Regulative Scaffold for Bone Defect Repair through Modulating Early Osteo-Immunomodulation, Middle Neovascularization, and Later Osteogenesis

Yuhao Yuan, Yan Xu, Yiyang Mao, Hongbin Liu, Minning Ou, Zhangyuan Lin, Ruibo Zhao, Haitao Long, Liang Cheng, Buhua Sun, Shushan Zhao, Ming Zeng, Bangbao Lu, Hongbin Lu, Yong Zhu and Can Chen**

Supporting figures:

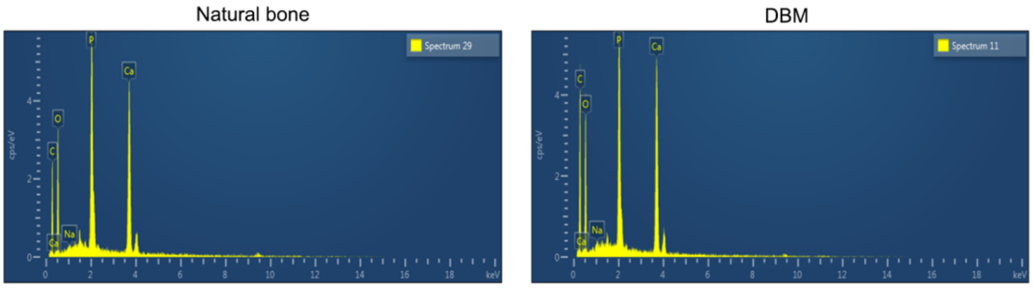

Spectrum 29				
Element	Line Type	Weight %	Weight % Sigma	Atomic %
C	K series	25.11	1.13	39.26
O	K series	33.56	1.01	39.39
Na	K series	0.22	0.18	0.18
P	K series	13.89	0.48	8.42
Ca	K series	27.22	0.71	12.75
Total		100.00		100.00

Spectrum 11				
Element	Line Type	Weight %	Weight % Sigma	Atomic %
C	K series	31.81	1.02	47.17
O	K series	31.20	0.91	34.73
Na	K series	0.25	0.16	0.20
P	K series	12.03	0.39	6.92
Ca	K series	24.71	0.60	10.98
Total		100.00		100.00

figure s1. Detection of Calcium (Ca) and Phosphorus (P) in DBM and natural bone using EDS.

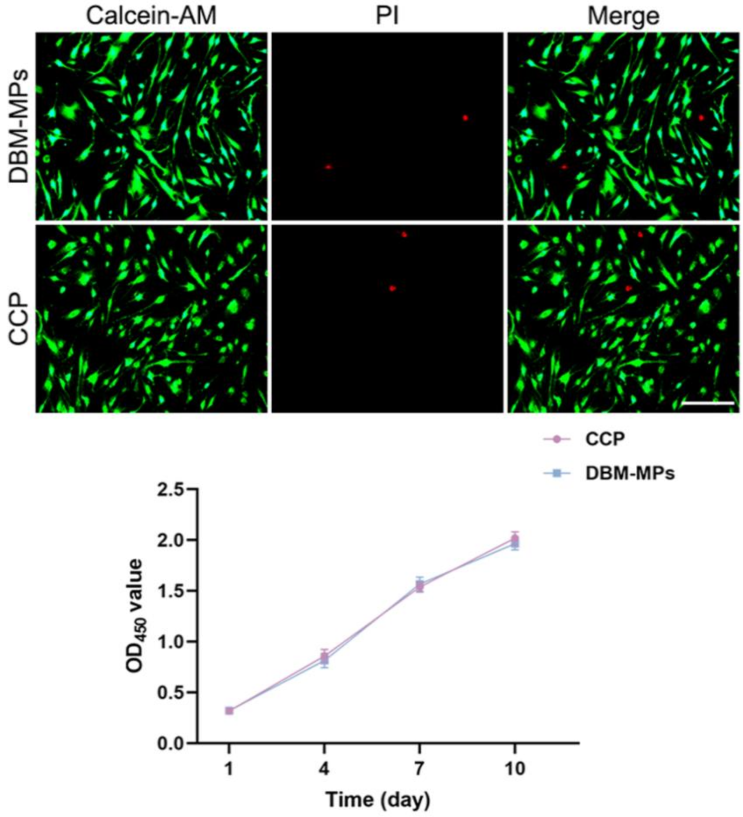

figure s2. CCK-8 and live/dead stain were used to assess the biocompatibility of DBM-MPs. Scale bar = 250 μm.

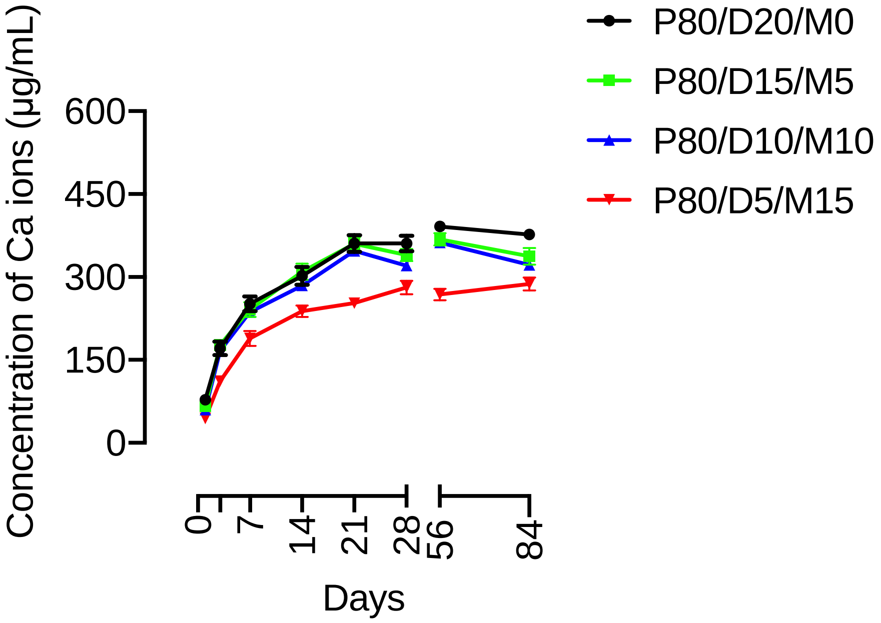

figure s3. In-vitro release curve of Ca ions from PLGA hybrid scaffolds.

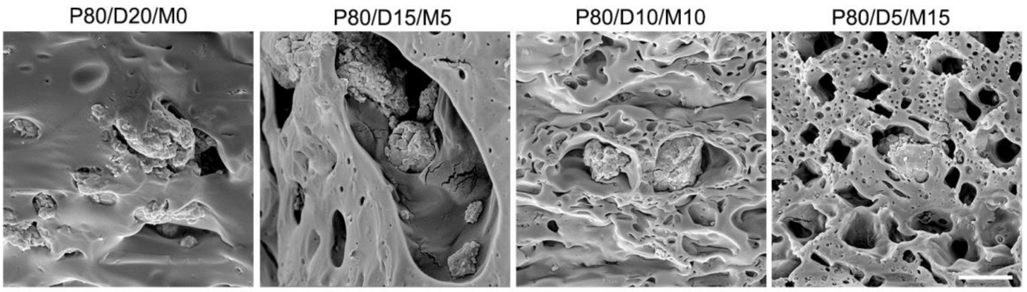

figure s4. Representative SEM images of the four PLGA hybrid scaffolds after 7 days degradation in-vitro. Scale bar = 25 μm.

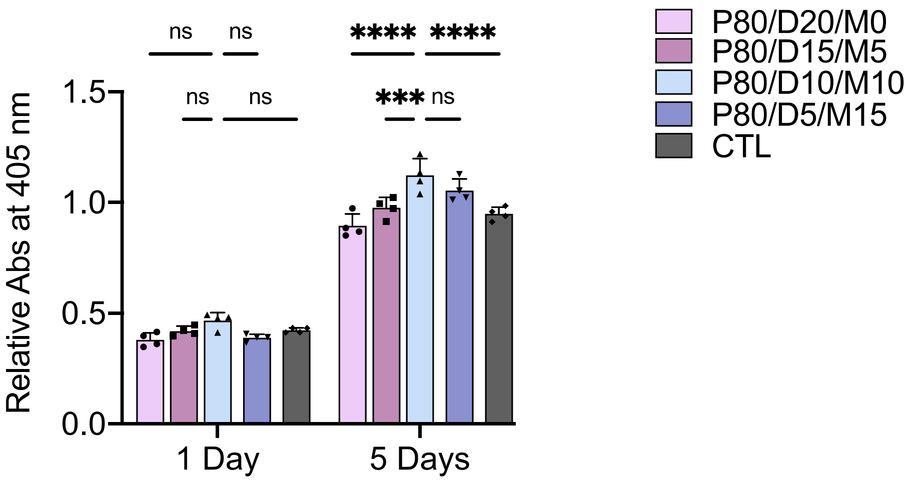

figure s5. CCK-8 assay showing proliferation of HUVECs on the four PLGA hybrid scaffolds at day 1 and 5.

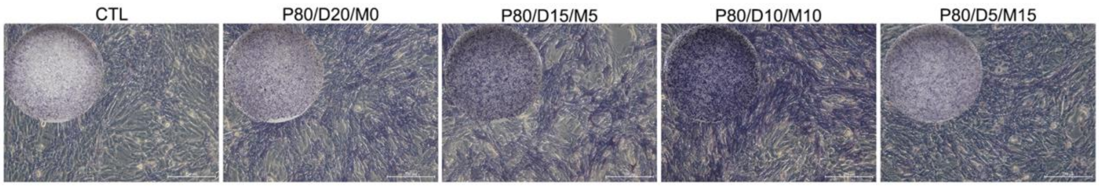

figure s6. ALP was used to assess the osteogenic differentiation of BMSCs under scaffold stimulation. After 7 days of osteogenic induction, ALP staining was performed. Scale bar = 250 μm.

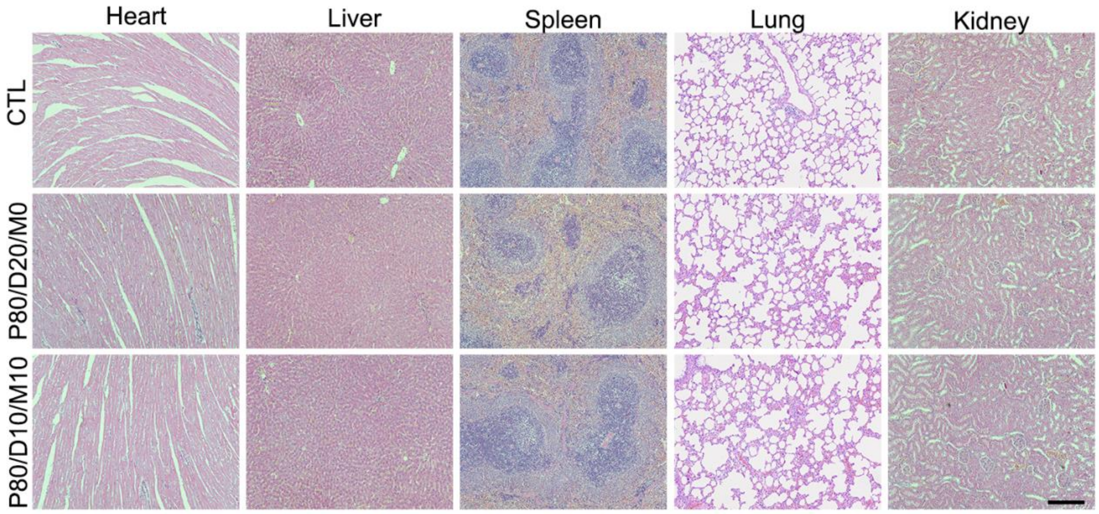

figure s7. The heart, liver, spleen, lung, and kidney tissue were taken and sliced at 12-week after scaffolds implantation to evaluate the systemic toxicity of the scaffolds. Scale bar = 200 μm.

Supporting table:

Table s1. Primer sequences

Primer name	Primer sequence (5' to 3')	Species
M-INOS Forward Sequence	TCACTCAGCCAAGCCCTCAC	Mouse
M-INOS Reverse Sequence	TCCAATCTCTGCCTATCCGTCTC	Mouse
M-CD86 Forward Sequence	AGCACTATTTGGGCACAGAGAAAC	Mouse
M-CD86 Reverse Sequence	GTGAAGTCGTAGAGTCCAGTTGTTC	Mouse
M-CD163 Forward Sequence	GGCAGTAGCAATATGTCTCCAACC	Mouse
M-CD163 Reverse Sequence	GATGGTGTCTTGTCTGAAGGTATGG	Mouse
M-CD206 Forward Sequence	TCTGGTGAACGGAATGATTGTGTAG	Mouse
M-CD206 Reverse Sequence	GCTTTGGTTGTAATGGATGAGTGTG	Mouse
R-Runx-2 Forward Sequence	TCGTCAGCGTCCTATCAGTTCC	Rat
R-Runx-2 Reverse Sequence	CTTCCATCAGCGTCAACACCATC	Rat
R-COL-1α Forward Sequence	GTGCGATGGCGTGCTATGC	Rat
R-COL-1α Reverse Sequence	CTATGACTTCTGCGTCTGGTGATAC	Rat
R-OPN Forward Sequence	AGCCATGAGTCAAGTCAGCT	Rat
R-OPN Reverse Sequence	ACTCGCCTGACTGTCGATAG	Rat
R-OCN Forward Sequence	CCTCACACTCCTCGCCCTATT	Rat
R-OCN Reverse Sequence	CCCTCCTGCTTGGACACAAA	Rat
M-MMP-9 Forward Sequence	CCTGTGTGTTCCCGTTCATCTTTG	Mouse
M-MMP-9 Reverse Sequence	TTATCCTGGTCATAGTTGGCTGTGG	Mouse
M-CTSK Forward Sequence	GGGTGTTCAAGTTTCTGCTGCTAC	Mouse
M-CTSK Reverse Sequence	ACTGCTTCTGGTGAGTCTTCTTCC	Mouse

R-CTSK Forward Sequence	ACTGCGACCGTGATAATGTGAAC	Rat
R-CTSK Reverse Sequence	TTATTCCGAGCCAAGAGAACATAGC	Rat
R-TRAP Forward Sequence	CTGGGTGGCTTCACATACGT	Rat
R-TRAP Reverse Sequence	GTAAGCCTCTGTGGTTCCCC	Rat
R-VEGF Forward Sequence	GAGCGGAGAAAGCATTTGTTTGTC	Rat
R-VEGF Reverse Sequence	CGCCTTGGCTTGTCACATCTG	Rat
R-PDGF-BB Forward Sequence	TTCCTGCCTCTCTGCTGCTAC	Rat
R-PDGF-BB Reverse Sequence	ATCTTCGTCTACGGAGTCTCTGTG	Rat
R-VACM-1 Forward Sequenc	CGTCTCATATTGGACATCTCTGCTG	Rat
R-VACM-1 Reverse Sequence	TGTTCACATAATCTGCTGGCATACC	Rat
R-CDH5 Forward Sequenc	AAGAACGAGGACAGCAACTTCAC	Rat
R- CDH5 Reverse Sequenc	GGAGATGAGCACAGGCAGGTAG	Rat
R- GAPDH Forward Sequenc	GTCCATGCCATCACTGCCACTC	Rat
R- GAPDH Reverse Sequenc	CGCCTGCTTCACCACCTTCTTG	Rat
M- GAPDH Forward Sequenc	GGCAAATTCAACGGCACAGTCAAG	Mouse
M- GAPDH Reverse Sequenc	TCGCTCCTGGAAGATGGTGATGG	Mouse
